# DFT-Machine Learning Joint Exploration of Transition Metal-Doped Ferroelectric BaTiO_3_ for Electrocatalytic Hydrogen Evolution

**DOI:** 10.1021/acsami.5c02406

**Published:** 2025-06-04

**Authors:** Haifa Qiu, Ming Yang, Haitao Huang

**Affiliations:** † Department of Applied Physics, 26680 The Hong Kong Polytechnic University, Hung Hom, Kowloon, Hong Kong 999077, China; ‡ Research Institute for Smart Energy, The Hong Kong Polytechnic University, Hung Hom, Kowloon, Hong Kong 999077, China; § Research Centre for Nanoscience and Nanotechnology, The Hong Kong Polytechnic University, Hung Hom, Kowloon, Hong Kong 999077, China

**Keywords:** DFT, machine learning, computational screening, transition metal doping, ferroelectric BaTiO_3_, electrocatalytic hydrogen evolution

## Abstract

Doping regulation holds promise to modulate electrocatalytic performance, yet it remains largely unexplored for ferroelectric (FE) BaTiO_3_ (BTO). By jointly employing first-principles calculations and machine learning (ML) analysis, we examine the effect of a broad range of transition metal (TM) doping in FE BTO on the electrocatalytic hydrogen evolution reaction (HER) activity and screen out the optimal TM dopants. We unveil that some early-to-middle group TM (V, Cr, Mo, Ta, Ru)-doped BTO surfaces feature higher synthesizability, which also exhibit noticeable HER activity with |Δ*G*_H*_| < 0.2 eV owing to intermediate hydrogen adsorption strength. Among all doped surfaces, the Mo-doped one shows optimal HER activity under both out-of-plane and in-plane polarization states. We reveal an intense interplay between the hydrogen adsorption configuration and the corresponding hydrogen bonding interaction, which relies more on the TM group than the polarization state. Most importantly, we propose a physically informed descriptor of the surface oxygen *p* band, which better describes HER activity trends of TM-doped surfaces than conventional band descriptors.This indicates the significance of the fractional filling and bandwidth of occupied oxygen *p*-band states. Moreover, we establish a robust ML model that can well predict HER activity with surface-independent input parameters alone with *R* ^2^ value above 0.93. From these parameters, we identify the inherent outer electron number of the TM dopant as the dominant feature, while the second ionization energy of the TM dopant and the initial polarization state show non-negligible feature importance. These findings could enlighten understanding, rational design, and accelerated discovery of element doping of FE materials for catalysis and other implications.

## Graphical abstract

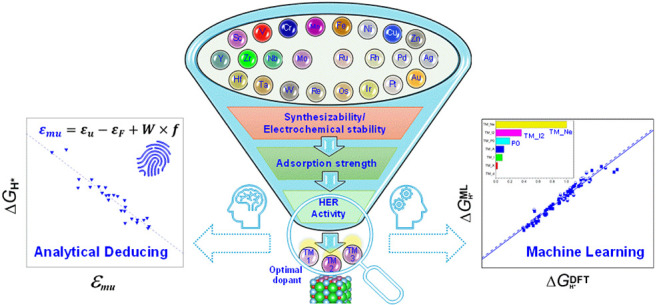

## Introduction

Electrocatalytic hydrogen evolution reaction (HER) from water splitting represents a promising way out for the efficient conversion and storage of intermittent renewable energy and a sustainable source of green energy carrier, which is pivotal for future energy safety and eco-balance. The pursuit of inexpensive and efficient HER catalysts, besides the commercial noble platinum-based catalysts, has not ceased in the past decades.^1–3^ Ferroelectric (FE) material-based catalysts, represented by BTO-based perovskites, have been in the spotlight of catalytic research in recent years due to their internal electric field and tunable polarization.^4–11^ However, bare FE BTO showcases unsatisfactory electrocatalytic HER performance because of its inherently low surface reactivity and electronic conductivity despite its low cost and eco-benignity. Hence modifications like elemental doping are often necessary.^12,13^

With advantages in compositional controllability, cost-effectiveness, experimental feasibility, and large-scale implementation prospects, elemental doping has been extensively exploited to improve electronic and/or ionic conductivity, regulate phase structure, modify active sites, and promote charge transfer to enhance catalytic performance.^14–17^ The way the catalyst is doped can be diverse, depending on the dopant type (metal or nonmetal), the occupying sites (A or B site of perovskite), the type of ion (cation, anion, or codoping of both),^18^ the doped region (bulk or surface),^16^ the majority carrier type (*n*- or *p*-type doping), the doping condition (high or low temperature, wet or dry, and so on), etc.^17^ All of these indicate the huge complexity yet vast possibilities of doping strategies. In recent years, much effort has been devoted to improving the catalytic performance of BTO by elemental doping at the A and/or B site and/or oxygen site, such as H,^19^ Na and/or Fe,^20^ Ce,^21^ Eu,^22^ Li and La,^23^ Co and La,^24^ Mo and Bi,^25,26^ and so on. For example, Maeda demonstrated that Rh doping in BTO gave rise to new visible light absorption bands and showed enhanced photocatalytic hydrogen evolution activity.^27^ Xie et al. studied the effect of Mo doping on the photocatalytic HER performance of BTO.^26^ Compared with bare BTO, a 2 at% Mo-doped BTO sample exhibited approximately a 200% increase in the hydrogen evolution rate to 63 mmol g^–1^ h^–1^.^26^ Harn et al. found that La- and Co-doped BTO nanoparticles showed notably enhanced oxygen evolution reaction activities compared to pristine BTO.^24^ Nonetheless, the doping regulation of FE BTO for electrocatalytic HER has been less studied.

Density functional theory (DFT)-based computational screening has been receiving increasing attention in recent years, emerging as a powerful auxiliary method not only to rationalize catalytic mechanisms but also to guide and accelerate the discovery of highly efficient catalysts, thereby sparing costly trial-and-error experiments.^28–36^ It has been demonstrated to be particularly effective in exploring the vast doping compositional configuration space.^28,33,37^ Li et al. studied, via DFT-based computational screening, the effect of transition metaldoping in CdS for photocatalytic hydrogen production, identifying Pt, Rh, and Pd as the ideal dopants in TM-doped CdS for photocatalytic HER.^32^ They further investigated the synergistic effect of codoping and found Co–Pt, Pd–Pt, and Co–Rh codoped CdS exhibiting remarkable HER catalytic activity, with significantly reduced |Δ*G*_H*_| values of less than 0.1 eV.^32^ Nørskov et al. presented a DFT-based high-throughput screening scheme that successfully used these strategies to recognize a new electrocatalyst of the BiPt binary alloy for HER among over 700 binary candidates whose activity was predicted to be comparable with or even outperform pure Pt, and experimental tests later confirmed this.^28^ Wexler et al. investigated the effect of surface nonmetal doping on the HER activity of Ni_2_P (0001)-terminated Ni_3_P_2_. Through DFT calculations, they found that both 2*p* nonmetals and heavier chalcogens provide optimal hydrogen adsorption. Using an ML algorithm, they revealed that the Ni–Ni bond length is dominant in describing HER activity via a chemical pressure-like effect.^37^ By DFT calculations, Zhou et al. screened a series of late 3*d* and 4*d* TM single atom-doped C_9_N_4_ monolayers as efficient electrocatalysts for water splitting and predicted that Co@C_9_N_4_ showed high electrocatalytic activity toward HER.^38^ However, the computational screening and understanding of TM-doped FE BTO for electrocatalytic HER remain lacking. The screening process generally involves several steps, including the initial setup of stability criteria for prescreening, the subsequent evaluation based on activity or selectivity descriptors, and the eventual identification of optimal catalysts or activity trends. In general, the underlying mechanism is unearthed to account for the doping effect and extract doping regulation principles for future research. Meanwhile, ML has been increasingly applied to probe activity origins or even to accelerate the DFT screening process.^37,39,40^

In this work, we studied the B-site doping of the TiO_2_-terminated FE BTO surface with a series of TM elements, ranging from 3*d* to 5*d* metals, through DFT-based computational screening and ML. We focused on surface doping with a single TM element. Moreover, the effects of out-of-plane and in-plane polarization directions on HER activity were taken into account during the screening process. The activity trends of doped BTO surfaces and the underlying structural and electronic origins were discussed.

## Methods

### Computational Details

First-principles calculations were performed using the Vienna Ab initio Simulation Package (VASP.5.4.4.18).^41^ Unless otherwise specified, the generalized gradient approximation (GGA) with the Perdew–Burke–Ernzerhof (PBE) functional was employed to describe the exchange-correlation effects, with ionic potentials treated using the projector augmented wave (PAW) approximation.^42–44^ A cutoff energy of 520 eV was used in all calculations, together with an electronic energy threshold of 10^–5^ eV and force criteria of 0.015 eV/Å to achieve convergence. The dispersion correction for van der Waals interactions between absorbates and substrates was adopted by using the DFT-D3 method from Grimme et al.^45^ Prior to surface calculations, bulk BTO with a tetragonal structure was relaxed. Out-of-plane upward/downward and in-plane polarized surfaces were modeled by BTO (001)/(00–1) and (100) surfaces, respectively, using *c*(2 × 2) slabs with three Pt layers as electron reservoirs, as mentioned in our previous work.^46^ The feasibility of implementing these polarization states has been demonstrated in experiments.^47,48^ The TiO_2_-terminated slabs contain 14 atomic layers, including three Pt layers, which were released during structural optimization yet fixed during hydrogen adsorption, and only the top two atomic layers were allowed to relax for the BTO surface. A 4 × 4 × 1 Monkhorst–Pack *k*-points mesh was used for integration in the Brillouin zone for BTO slab supercells.^49^ A 15 Å thick vacuum layer was added for all slabs to avoid spurious interactions. Dipole correction was set for all slab calculations. The solvent effect was considered for surfaces with optimal dopants using an implicit solvation model.^50,51^ To deal with the inability of the Berry phase method for the noninsulating systems,^52^ we evaluated the “effective polarization” values of doped BTO using the equation below (eq 1):^53^

1 $$\Delta P_{\mathrm{eff}}=\frac{e}{\Omega }\underset{i}{\sum }Z_{i}^{*}d_{i}$$

where *e* is the electronic charge, Ω is the unit cell volume, $Z_{i}^{*}$ is the Born effective charge (BEC) of atom *i*, and *d*_*i*_ is the displacement along the polarization direction relative to the nonpolar phase. We considered only the displacement and polarization along the direction normal to the surface. The BEC values used for barium, titanium, and oxygen were +2.83, + 5.81, and −4.73, respectively.^54^ This resulted in a polarization value of 34.0 μC/cm^2^ for undoped bulk BTO, close to the value of 30.6 μC/cm^2^ computed by using the Berry phase method. For transition metal-doped BTO, we set the BEC of the Ti atom for the dopant atom. The polarization calculated herein reflects the extent of FE structural distortion occurring within the system instead of the true FE polarization calculated by the Berry phase method. In this work, we use the term “effective polarization” to denote this kind of polarization regardless of its reversibility.

## Results and Discussion

### Modeling and Computational Screening Workflow

Like our previous work,^46^ we chose the TiO_2_-terminated surface for study due to its aqueous stability in acidic conditions. The *c*(2×2) slab surface model was chosen based on a balance between computational demands and effective physical-chemistry insights. By a similar dopant coverage as reported,^55,56^ in our model, half of the Ti atoms on the surface are substituted by the transition metal dopant, as shown in Figure 1a–c. For the sake of eco-benignity, we excluded those dopant candidates with high toxicity or radioactive hazards, such as Cd, Hg, and Tc. The workflow of computational screening is shown in Figure 1d, which consists of three parts. The first is the evaluation of the thermodynamic and electrochemical stability of doped BTO surfaces. The second goal is to screen for the most thermodynamically stable adsorption configuration. The last is to identify the HER activity trend with polarization states considered and conduct a mechanism study.

**Figure 1:**
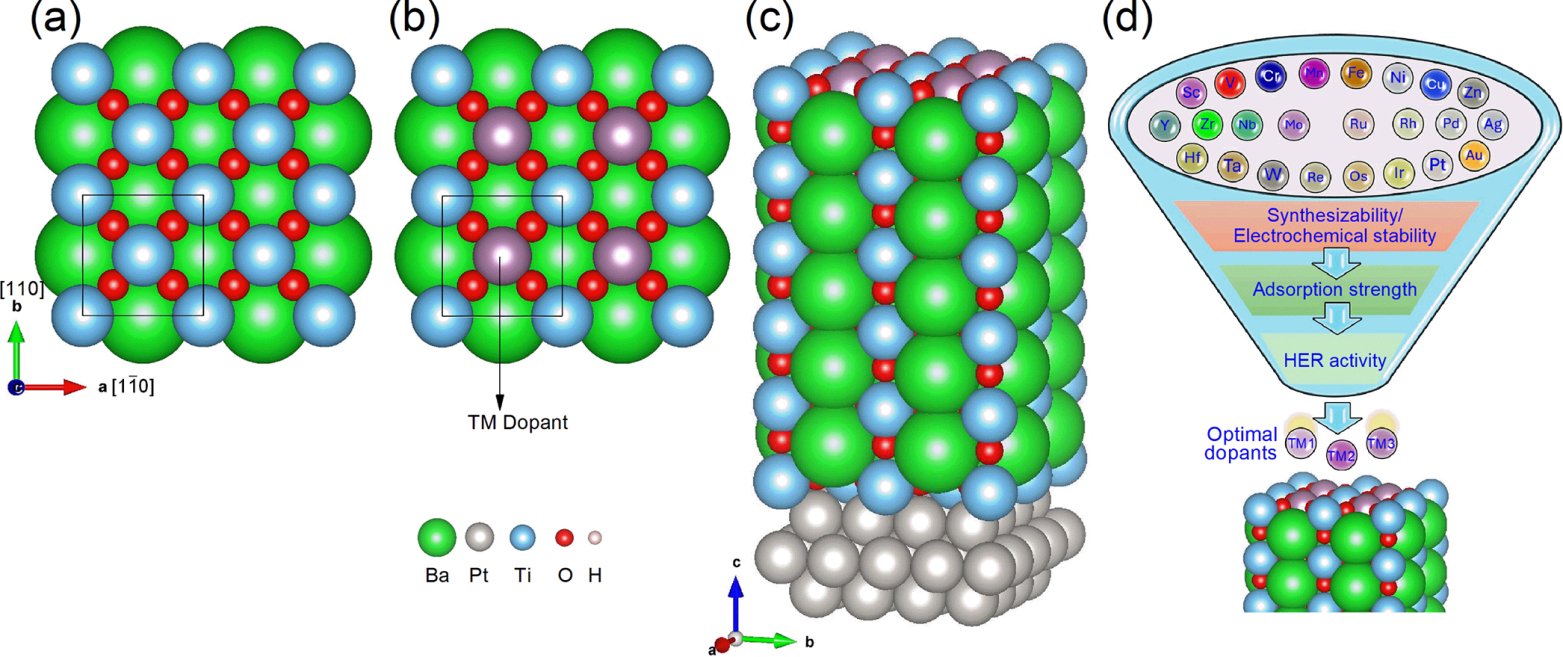
Atomic structures of (a) undoped BTO viewed from the [001] direction, (b) doped BTO viewed from the [001] direction, and (c) 3D view. (d) Schematic illustration of screening workflow.

### Evaluation of the Synthesizability and Electrochemical Stability of TM-Doped BTO

To begin with, we examined the formation energy of doped BTO with reference to the undoped one to evaluate the relative stability of doping, which usually reflects the synthesis ability of doped materials. The relative formation energy of substituting Ti with a dopant is defined by the following equation (eq 2):^57^

2 $$E_{\mathrm{form}}=E_{\mathrm{BTO}}^{\mathrm{TM}}-\left[E_{\mathrm{BTO}}^{B}+\mu_{\mathrm{TM}}-\mu_{\mathrm{Ti}}\right]$$

where $E_{\mathrm{form}}$ denotes the substitutional formation energy per dopant, $E_{\mathrm{BTO}}^{\mathrm{TM}}$ and $E_{\mathrm{BTO}}^{B}$ refer to the total energies of the doped and undoped BTO slabs, respectively, while $\mu_{\mathrm{TM}}$ and $\mu_{\mathrm{Ti}}$ denote the chemical potentials of TM and Ti in their bulk states, respectively. To avoid the precipitation of undesired secondary transition metal solids, oxides, or gas evolution, the chemical potentials of the constituent species in the atomic reservoir need to satisfy some boundary conditions, as described by the equations below (eqs 3− [Disp-formula eq9]):

3 Δμ(Ti)+Δμ(Ba)+3Δμ(O)=ΔH(BTO)

4 Δμ(TM)≤0

5 Δμ(Ti)≤0

6 Δμ(Ba)≤0

7 Δμ(O)≤0

8 $$\Delta \mu \left(\mathrm{Ti}\right)+2\Delta \mu \left(O\right)\leq \Delta H\left({\mathrm{TiO}}_{2}\right)$$

9 Δμ(Ba)+Δμ(O)≤ΔH(BaO)

where Δμ(i) is the chemical potential of constituent species *i* (*i* = Ba, Ti, TM, O), relative to its elemental standard state, i.e., $\Delta \mu \left(i\right)=\mu \left(i\right)-\mu^{0}\left(i\right)$ . $\mu^{0}\left(i\right)$ is approximated by the total energy of constituent species, E(i) , with $\mu^{0}\left(O\right)=1/2E\left(O_{2}\right)$ . ΔH(j) refers to the formation enthalpy of the solid oxide compound *j* (*j* = BaO, TiO_2_, BTO). Chemical potentials are constrained based on the thermodynamic laws of equilibrium. The formation enthalpy in solid-state physics is defined as the energy difference between the compound product and the energy sum of the constituent elements. For simplicity, we assume TM is rich in the atomic reservoir so that Δμ(TM) equals zero. As a result, $\mu_{Ti}$ will change with the evolution of the oxygen chemical potential μ(O) . The *E*_form_ characterizes the relative thermodynamic proneness of substitutional doping of TM on the BTO surface. When *E*_form_ is negative, it means that TM is thermodynamically feasible to substitute Ti on the TiO_2_-terminated BTO surface. In other words, the synthesis of doped BTO is favorable. Instead, when it is positive, the Ti on the surface TiO_2_ layer is less thermodynamically favorable to be substituted by the TM, and its synthesis can be difficult.

First, we calculated the formation enthalpies of oxides, which are comparable to experimental values and other calculated results, as shown in Table 1. The evolution of the stable region for the formation of BTO with varied oxygen chemical potential is shown in Figure S1. We can see that the feasible chemical potential of Ti is dependent on the oxygen potential. The chemical potential of Ti for the stable formation of BTO increases as the oxygen chemical potential decreases to −5.5 eV, with Ba and Ti being likely to precipitate. Accordingly, the chemical potential of Ti and O can only be tunable in an appropriate range (for O: −5.5 ∼ 0 eV and corresponding Ti:0 ∼ −10 eV).

**Table 1:** A Comparison of Experimentally Measured and Calculated Formation Enthalpies

Oxide	Experimental^58^/eV	Present work/eV	Previously calculated^59–61^ /eV
BTO	–17.2	–16.0	–16.3
TiO_2_	–9.79	–9.39	–8.44
BaO	–5.74	–5.19	–5.19

The formation energy tends to be more negative under higher oxygen chemical potential, i.e., the TM dopants are more likely to be doped on BTO. For example, in an oxygen-rich atmosphere with Δμ_O_ = 0 eV, almost all the formation energies are negative, as shown in Figure 2a, implying the possibility of substitutive doping for most of the TM dopants. Given the same chemical potential, the formation energy shows an apparent increasing trend with the dopant atomic number for each group of 3*d*, 4*d*, and 5*d* TM elements. As the oxygen chemical potential is lowered to, say, Δμ = −2 eV, the formation energy of the mid -to-late TM dopants turns positive, as shown in Figure S2. This indicates that early group TM elements are thermodynamically more favorable for the surface doping of BTO and hence exhibit higher synthesizability compared with the mid-to-late ones. An oxygen-rich atmosphere is conducive to the TM doping of BTO. In a very oxygen-poor atmosphere, the formation energy is positive for all TM-doped BTO, as shown in Figure S2. This implies that the oxygen chemical potential should be carefully controlled for the successful synthesis of doped BTO. For the correlation of oxygen chemical potential with temperature and partial pressure in experiments, one may refer to this work.^62^ The synthesizability of TM-doped BTO systems is supported by the grand potential phase diagrams of ternary oxides at finite temperature using Pymatgen,^63,64^ as shown in Figure S3 and the literature survey of doping experiments,^26,65–70^ regardless of polarization states and dopant stoichiometry.

**Figure 2:**
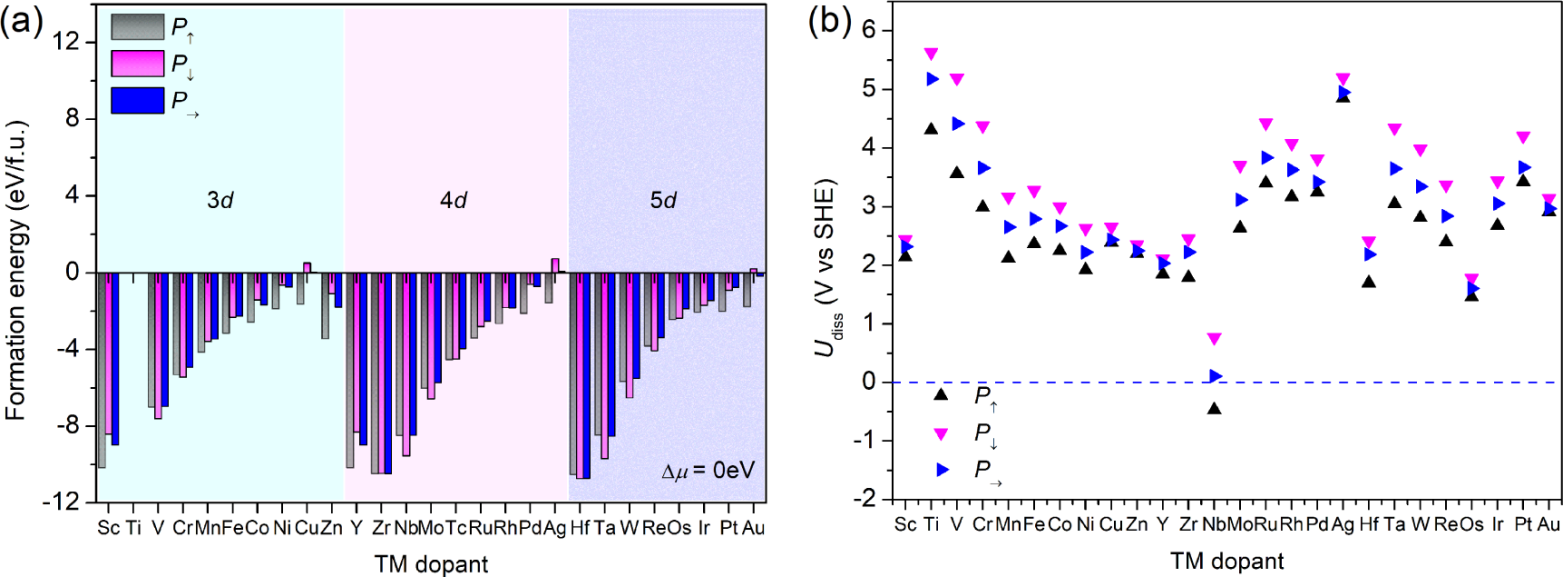
Evaluation of the thermodynamic and electrochemical stability of TM-doped BTO. (a) The formation energy and (b) dissolution potentials of TM-doped BTO under various polarization states.

In analogy to the estimation of the dissolution of elements from alloys, we also examined the dissolution potential of TM dopants in doped BTO, which was reported to reflect the electrochemical stability, according to the equation below (eq 10):^71,72^

10 $$U{\left(\mathrm{TM}\right)}_{\mathrm{diss}}=U_{0}+\left(E-E\left(BTO\_TM\right)+E\left(BTO\_vac\right)\right)/ne$$

where *U*_0_ refers to the dissolution potential of the TM dopant at standard state, *E* refers to the total energy of TM in its bulk, *E*(BTO_TM) refers to the total energy of the TM-doped BTO, *E*(BTO_vac) refers to the total energy of BTO with a Ti vacancy, and *n* is the number of electrons transferred during dissolution. Materials with *U*_diss_ > 0 V vs SHE are regarded as electrochemically stable. As presented in Figure 2b, we can see that all of these doped materials show positive dissolution potential values except for Nb-doped BTO in an upward polarization state, indicating that most TM-doped BTOs are stable in standard electrochemical environments, irrespective of polarization states.

### Effect of TM Doping on the Structural and Electronic Properties of FE BTO

To understand the effect of doping on structural and electronic properties, we compared the effective polarization Δ*P*_eff_ and the density of states (DOS) of the TM-doped BTO surface. The effective polarization is calculated based on the Born effective charge (BEC) and the ionic shift relative to the nonpolar reference phase.^53,54,73^ It is shown in Figure S4a that all of the Δ*P*_eff_ values oscillate in a similar pattern with the TM dopant for out-of-plane and in-plane polarization states. The absolute Δ*P*_eff_ value for each TM dopant follows the order: *P*_↓_ > *P*_↑_ > *P*_→_, implying the overall out-of-plane FE distortion trend. The average Δ*P*_eff_ value for upward, in-plane, and downward polarization states is 31.1, −4.1, and −39.2 μC/cm^2^, respectively. It turns out that the polarization state has a varied and profound impact on structural reconstruction, but TM doping will not change the overall trend of effective polarization with the polarization state. As presented in Figure S4b–d, in most cases, valence or conduction bands spread across the Fermi level, implying that electronic (acceptor or donor) doping occurs upon TM doping. Accordingly, the midgroup TM shows a higher density of states at the Fermi level. The enhanced occupation of electronic states at the Fermi level upon TM doping is believed to be conducive to improving the electronic conductivity of electrocatalysts and hence catalytic performance. The above results show that TM doping has a significant influence on the structural and electronic properties of the BTO surface, which may affect its electrocatalytic activity.

### Evaluation of TM Doping on Hydrogen Adsorption and HER Activity

Doping may increase the number of surface adsorption sites for hydrogen due to the introduction of heteroatom dopants and the reduced surface symmetry. On the other hand, the lowered surface symmetry complicates and greatly expands the surface adsorption configurational space, as exemplified in Figure S5, making it challenging for DFT screening owing to largely increased computational demands. Considering both the out-of-plane and in-plane polarization states, the magnitude of the entire configuration space for TM-doped BTO surfaces reaches above 1,000, which requires considerable effort and time in DFT-level computational screening. Although hydrogen can possibly be adsorbed on all of these sites, the whole hydrogen evolution process is subject to the overall limiting potential barrier during hydrogen adsorption/desorption, which requires the adsorption energy to be negative for the initial hydrogen adsorption to occur. During computational screening, it is necessary to search globally for the most thermodynamically stable site for each doped BTO surface, on which the rate-limiting step occurs, ensuring the subsequent hydrogen desorption process during the HER.

The most stable surface configuration depends more on the TM group than on the polarization state. There are three major stable hydrogen adsorption configurations, as presented in Figure 3a–c. The first one is the surface oxygen that is adjacent to the TM dopant for all 3*d* TM-doped BTO under downward polarization states, as shown in Figure 3a. The second is the hollow site over Ba, as shown in Figure 3b. As presented in Figure 3c, the third one is near the top site of TM for most early-to-mid group 5*d* dopants. The distribution of the most stable adsorption configuration with polarization state and TM group is shown in Figure 3d.

**Figure 3:**
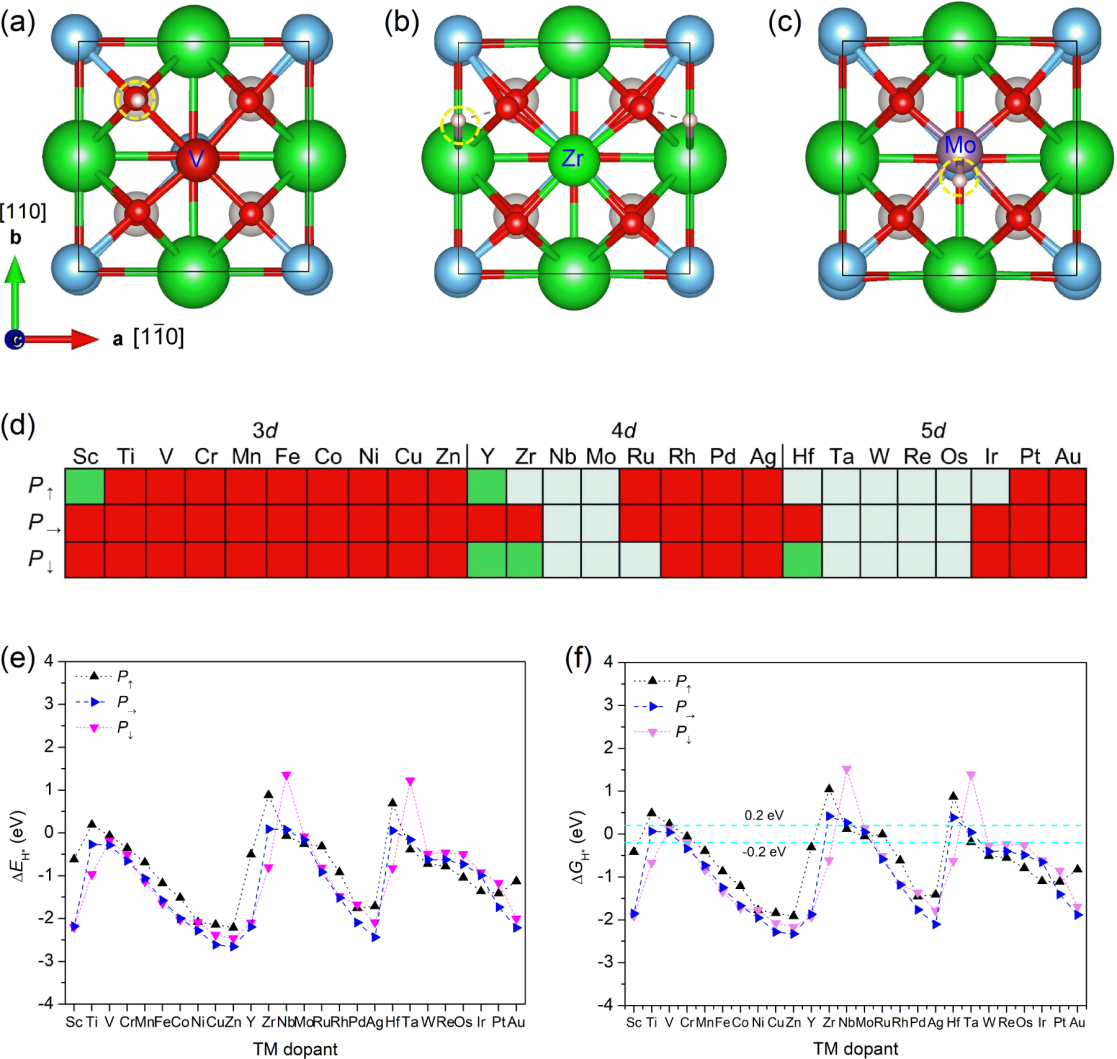
Stable hydrogen adsorption configurations and Gibbs free energy of hydrogen adsorption, Δ*G*_H*_, on the TM-doped BTO surface. (a) Hydrogen adsorbing on the surface oxygen site adjacent to TM, (b) hydrogen adsorbing on the hollow site over Ba in the vicinity of surface oxygen, and (c) hydrogen adsorbing near the on-top site of TM dopant. Ba, Ti, O, H atoms are in green, blue, red, and white, respectively. The yellow circle indicates the hydrogen adsorption region. (d) Distribution of stable hydrogen adsorption configuration for various TM dopants under different polarization states, in which the red rectangle corresponds to Figure 3a, the green one corresponds to Figure 3b, and the light blue one corresponds to Figure 3c. (e) The adsorption energy, Δ*E*_H*_, and (f) Gibbs free energy change of hydrogen adsorption, Δ*G*_H*_, on the most stable site of TM-doped BTO surfaces.

The most thermodynamically stable adsorption energy for each dopant under varied polarization states is shown in Figure 3e. It is clearly observed that there is a decreasing energy trend (increased adsorption strength) with the TM atomic number in each *d*-metal group in the periodic table for all polarization states, except for the first several elements. For some mid-group 3*d* and 4*d* TM dopants, Δ*G*_H*_ values are similar under downward and in-plane polarization states, compared with the upward polarization state. This suggests that the TM-doped surface tends to show varied responses toward different polarization states. All of these manifest that the polarization effect is non-negligible upon TM doping and varies with the TM group.

Then, the Gibbs free energy change of hydrogen adsorption, Δ*G*_H*_, at pH 0 under standard conditions was also compared for each dopant, as shown in Figure 3f. We chose the free energy range between −0.20 and 0.20 eV as the screening criterion for HER activity,^74^ given that a slight increase in pH may lead to a slight negative shift of Δ*G*_H*_. We also perceived a similar trend with the TM element order in the periodic table for Δ*G*_H*_; namely, Δ*G*_H*_ decreases as the atomic number increases in each *d*-metal group. Consequently, there exists a diagonal relationship for Δ*E*_H*_ and Δ*G*_H*_ among the 3*d*, 4*d*, and 5*d* groups.^75^ For example, the Δ*G*_H*_ values of V-doped BTO are approximately equal to those of Mo-, Fe-, and Rh-doped ones. This trend intensively implies that the strength of hydrogen adsorption and the corresponding free energy change can be closely linked to certain intrinsic atomic-number-dependent but periodically changed properties of TM dopants. As a result, for upward polarization, 5 dopants fall within the criteria: Cr (−0.06 eV), Mo (−0.05 eV), Ru (−0.01 eV), and Ta (−0.19 eV) (Nb (0.12 eV) is excluded due to electrochemical instability). For downward polarization, there are 2 qualified dopants: V (0.11 eV) and Mo (0.13 eV). For in-plane polarization, there are 3 qualified dopants: V (0.04 eV), Mo (0.04 eV), and Ta (0.03 eV). Noteworthily, the Mo dopant shows an optimal Δ*G*_H*_ value under all these polarization states. We also surveyed the solvent effect for surfaces with these optimal dopants (V, Cr, Nb, Mo, Ru, and Ta) by imposing the implicit solvation model. We found that the difference in Δ*G*_H*_ for most surfaces with and without the solvation model is within 0.10 eV (see Table S1), suggesting a minor solvent effect on the hydrogen binding of these doped surfaces. Thus, for the majority of these surfaces, the HER activity remains within the optimal region. Importantly, Mo remains the optimal dopant under both out-of-plane and in-plane polarization states. The dissolution potential trends for optimal dopants on doped surfaces can be combined with the Pourbaix diagrams of TM-doped TiO_2_ at applied potential and pH (as shown in Figure S6) for more practical electrochemical implications. For *p*(2 × 2) surface (see Figure S7) with reduced surface coverage of TM dopants, i.e., one-quarter of surface Ti atoms are substituted by Mo, the values of Δ*G*_H*_ under out-of-plane and in-plane polarization states are still within or near the optimal region for HER, as shown in Table S2.

### Mechanism Understanding of Electronic and Structural Surface Factors for HER Activity of TM-Doped FE BTO

To gain insights into the underlying mechanism of hydrogen adsorption strength and HER activity upon doping, the electronic structure and bonding of the TM-doped BTO surface were analyzed. First, the crystal orbital Hamilton population (COHP) method was used to evaluate the bond strength for typical hydrogen adsorption configurations with the downward polarization state. The COHP method can resolve the band structure energy into antibonding, nonbonding, and bonding contributions with localized atomic basis sets.^76^ The energy integration of the COHP curve for a pair of atoms up to the Fermi level (ICOHP) was reported to reflect the bond length and bond covalency.^77–79^ We unveil that the bonding and antibonding interaction largely relies on the different hydrogen adsorption configurations. For instance, in the case of V doping, the hydrogen adsorbs on the oxygen site adjacent to the V dopant, as shown in Figure 3a, and as shown in Figure 4a, a strong bonding peak below the Fermi level at approximately 9 eV is ascribed to H1*s*-O2*pz* coupling. The ICOHP value for the O–H bond, with a bond length of 0.974 Å and near-vertical hydrogen adsorption configuration, is −7.74 eV, signaling noticeable covalency. For the hydrogen adsorption over the hollow site adjacent to surface oxygen, such as that shown in Figure 3b, the O–H bond exhibits a longer bond length of 1.2 Å. The bonding states are mainly contributed by the H1*s*-O2*px* orbital interactions instead of the O2*pz* orbital interactions due to the hydrogen bond tilting in plane and aligning with the O2*px* orbital. As a result, its ICOHP value is −3.70 eV in Figure 4b, suggesting a largely reduced covalency strength. For the Ba–H bond in Figure 4c, the major bonding contribution is from the Ba6*s*-H1*s* orbitals. The Ba–H displays a large bond length of 2.85 Å, and the corresponding ICOHP is −0.05 eV, implying rather weak covalency, possibly being dispersion or mainly ionic interaction, which can be negligible. For the Mo-doped BTO, the single Mo–H bond of 1.72 Å has an ICOHP of Mo–H of −1.56 eV, as shown in Figure 4d, implying mild covalency. The antibonding interaction mainly results from H1*s*-Mo4*dz* ^2^ orbitals, while the bonding interaction majorly arises from H1*s*-Mo4*dyz* orbitals in addition to the dominant H1*s*-Mo4*dz* ^2^ orbitals. Likewise, this can also be explained by its on-top hydrogen adsorption geometry, which favors the TM *dz* ^2^ orbital interaction with hydrogen. Due to these varied antibonding and bonding interactions, the charge transfer from surface oxygen or dopant to hydrogen also varies. According to the Bader charge analysis, we disclose that the charge gain of hydrogen upon adsorption increases from the Nb-doped surface (0.34 *e*), V-doped surface (0.50 *e* per H), Zr-doped surface (0.68 *e* per H), to the Mo-doped surface (0.76 *e* per H), as presented in Figure 4e. The charge transfer to hydrogen is higher on doped surfaces with optimal HER activity, similar to the reported trend.^80^ We studied the trend of Δ*G*_H*_ with ICOHP for the 3*d* TM-doped BTO surface under the downward polarization state, as shown in Figure S8. We observe a noticeable linear correlation between ICOHP and Δ*G*_H*_, but the correlation is not significant for some late-group TM dopants.

**Figure 4:**
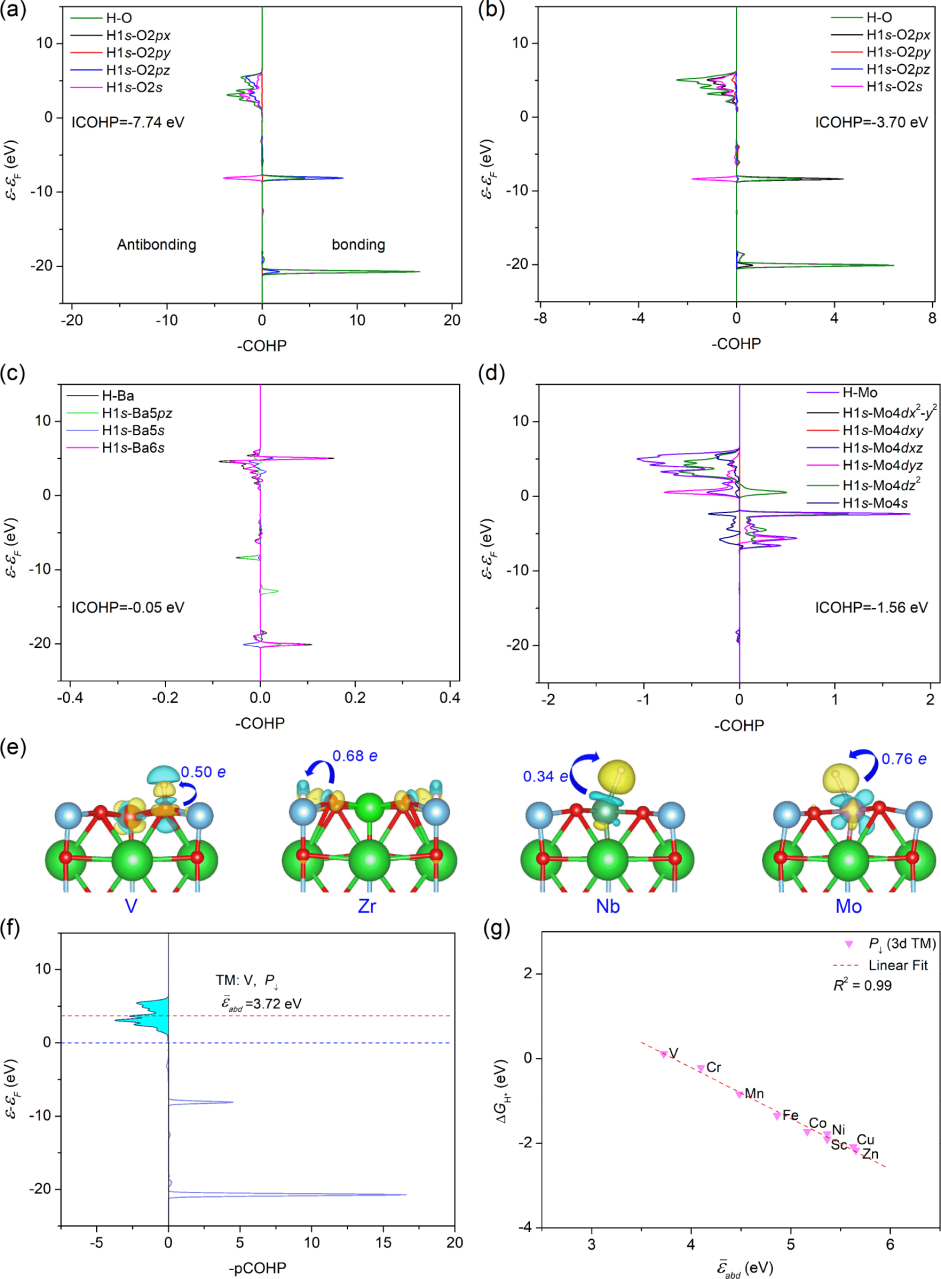
Calculated orbital-resolved projected crystal orbital Hamilton population (COHP) for the H–O/Ba/TM bond on the (a) V-doped, (b,c) Zr-doped, and (d) Mo-doped BTO surface with the downward polarization state (*P*↓). The zero energy is set at the Fermi level. (e) Charge density difference plots and charge transfer from oxygen or TM to hydrogen on typical TM-doped BTO surfaces (TM = V, Zr, Nb, Mo) with isosurface level of 0.008 *e*/Bohr.^3^ Yellow regions indicate charge gain, while blue regions indicate charge depletion. (f) An example of projected COHP (pCOHP) of O–H bond on V-doped BTO and its antibonding energy center, with cyan area indicating the antibonding states. (g) Gibbs free energy change of hydrogen adsorption, Δ*G*_H*_, vs antibonding energy center, ${\mathrm{\varepsilon ̅}}_{\mathrm{abd}}$ , for 3*d* TM-doped BTO surface. The linear regression does not include Ti.

To reveal the origin behind the varied adsorption strength for TM-doped BTO at the very adsorbing state, we characterize the evolution of Δ*G*_H*_ with the energy center of antibonding states of COHP, which has been demonstrated to be intimately related to the hydrogen adsorption strength.^81^ The antibonding energy center is defined by the below equation (eq 11) in analogy to the method used for *d*-band centers:

11 $${\mathrm{\varepsilon ̅}}_{\mathrm{abd}}=\frac{\int_{0}^{\varepsilon_{\max}}\xi \left(\varepsilon \right)\mathrm{\varepsilon d\varepsilon}}{\int_{0}^{\varepsilon_{\max}}\xi \left(\varepsilon \right)\mathrm{d\varepsilon}}$$

where ε corresponds to the energy of the COHP state and ξ*(*ε) refers to the crystal orbital Hamiltonian population (COHP) of orbital pair contributions at a given energy ε . ε̅_*abd*_ denotes the averaged energy of the unoccupied antibonding energy curve, as depicted in Figure 4f. ε_max_ refers to the upper energy limit of the COHP of interest, which we set to be 15 eV here.

As shown in Figure 4g, we can see a strong negative linear correlation between ε̅_abd_ and Δ*G*_H*_ with a high correlation coefficient of above 0.99. According to the correlation, the higher the ε̅_abd_, the more negative the Δ*G*_H*_ value. The ε̅_abd_ could reflect the amount of energy required for antibonding electron transfer; that is, it measures the average potential energy required for originally occupied antibonding electrons of the surface and the hydrogen, before hydrogen adsorption, to be transferred to the Fermi level during surface-hydrogen orbital coupling. As shown in Figure S9, under each polarization state, the Δ*G*_H*_ value of TM-doped BTO inversely changes with ε̅_abd_ for each TM group and the Δ*G*_H*_ gradually declines as ε̅_abd_ rises. Notable linear correlation still exists, especially for in-plane and downward polarization states. It turns out that the average antibonding center of the COHP is a relevant direct fingerprint of surface bonds and orbitals to describe the hydrogen adsorption strength for TM-doped surfaces despite varied hydrogen adsorption configurations.

So far, we have discussed the underlying roots of the adsorption trend of hydrogen at the atomic orbital level for the TM-doped BTO surface upon hydrogen adsorption. For computational screening, it is quite important to find the fingerprints from the TM-doped BTO surface before hydrogen adsorption to explain the surface reactivity and capture the adsorption strength trend, which can help accelerate the identification of effective catalysts for future development. We have tried the oxygen 2*p* band as has been used in our previous work.^46^ We began with the evaluation of the surface energy bands. The TM *dz* ^2^ contributes mainly to the conduction band of surface states, while the O2*p* contributes mainly to the valence band of surface states. Therefore, we choose the lower parts of PDOS for the study of the evolution of O2*p* band properties such as the band center and band edge. The band edge proposed by Vojvodic et al. is given by the following eq (eqs 12 and 13):^82^

12 $$\varepsilon_{u}={\mathrm{\varepsilon ̅}}_{p}+\frac{W}{2}$$

13 $$W={\left(\frac{\int_{\varepsilon_{\min}}^{\varepsilon_{\max}}\rho \left(\varepsilon \right){\left(\varepsilon -{\mathrm{\varepsilon ̅}}_{p}\right)}^{2}d\varepsilon }{\int_{\varepsilon_{\min}}^{\varepsilon_{\max}}\rho \left(\varepsilon \right)d\varepsilon }\right)}^{\frac{1}{2}}$$

where *ε*_*u*_ refers to the upper band edge, ε̅*_p_* refers to the oxygen *p*-band center, as described in our previous work,^46^ which characterizes the band position, *W* refers to the bandwidth that characterizes the band shape, ε_max_ and ε_min_ refers to the upper and lower limit of the band under study, which we set to be 2 eV and −10 eV, respectively. ρ (ε) refers to the density of states at energy ε. As a result, we find that Δ*G*_H*_ exhibits similar trends with the change of the upper band edge and band center of O2*pz*, that is, Δ*G*_H*_ decreases with the change of ε*_u_* and ε̅_*p*_, as shown in Figure S10. It shows that surface oxygen with too high-lying band center and edge tends to absorb hydrogen strongly, while one with too low-lying band center and edge loosely absorbs or fails to absorb hydrogen. It can be observed that the negative linear correlation between Δ*G*_H*_ and band edge (Figure S10d–f) is stronger than that with band center (Figure S10a–c), with *R* ^2^ (the coefficient of determination) improved by 8%, 6%, 6% for upward, in-plane, and downward polarization states, respectively. This suggests that the HER activity depends more on the band edge. This indicates that the difference made by the bandwidth is not insignificant. The strong band peak displays narrow bandwidth while the weak band peak displays wide bandwidth. This may lead to the formation of strong or weak σ bond, respectively, via coupling between the adsorbent surface oxygen 2*p* orbital and the 1*s* orbital of hydrogen adsorbate upon hydrogen adsorption. This may account for the better description of the activity trend by the band edge relative to the band center in the present study. To search for other possible descriptors that may enhance the description, we made a series of attempts through combinatorial enumeration from different surface electronic factors such as work function, vacuum level, Fermi energy, band center, band filling, bandwidth, and so on. Eventually we find a novel descriptor, which is constructed based on the band edge modification by considering the oxygen *p*-band fractional filling below the Fermi level, and it is defined as below equations (eqs 14 and 15):

14 $$\varepsilon_{\mathrm{mu}}=\varepsilon_{u}-\varepsilon_{F}+W\times f$$

$$f=\frac{\int_{\varepsilon_{\min}}^{\varepsilon_{F}}\rho \left(\varepsilon \right)\mathrm{d\varepsilon}}{\int_{\varepsilon_{\min}}^{\varepsilon_{\max}}\rho \left(\varepsilon \right)\mathrm{d\varepsilon}}$$ 15

where ε_mu_ refers to the modified upper band edge and ε_**F**_ refers to the Fermi energy. *f* refers to the fractional filling of *p*-band states up to the Fermi level. The third term in eq 14 can be physically interpreted as the normalized amount of the occupied *p-* band states or electrons. Figure 5a illustrates different band descriptors. The evolution of Δ*G*_H*_ with the change of the modified band edge is shown in Figure 5b–d. We observe a significantly enhanced linear negative correlation under each polarization state relative to the case of band edge, as indicated from *R* ^2^ heightened by 11% and 4% for upward and downward polarization states, respectively. In contrast to the case of the band center, the *R* ^2^ has largely increased by 19%, 6%, and 10% for upward, in-plane, and downward polarization states, respectively. From the negative correlations of linear regression between Δ*G*_H*_ and ε_mu,_ a positive shift in ε_mu_ upon TM doping will give rise to stronger surface adsorption.

**Figure 5:**
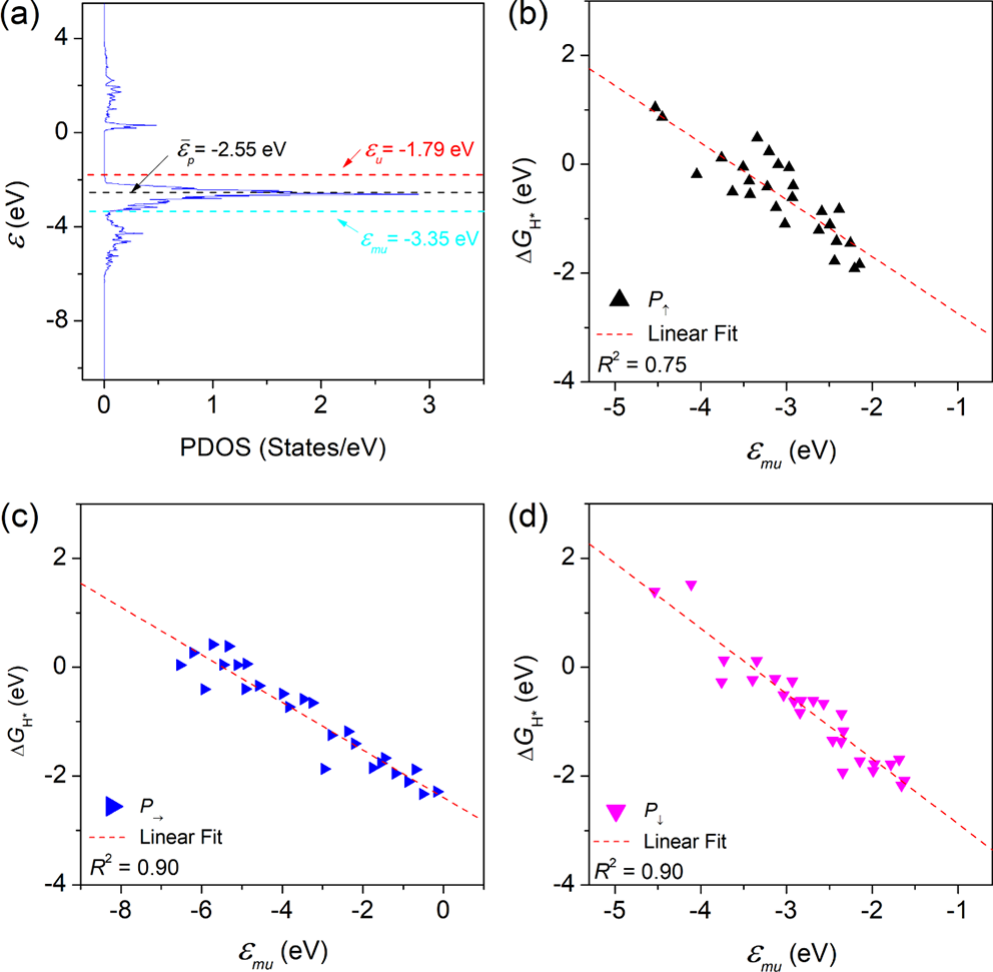
(a) A comparison of the O*pz* band and its band center (ε̅_*p*_), band edge (*ε*_*u*_), and modified band edge (ε_*mu*_) for V-doped BTO under downward polarization state. The correlation of Gibbs free energy change of hydrogen adsorption, Δ*G*_H*_, with the modified upper band edge of surface oxygen 2*pz* band, $\varepsilon_{\mathrm{mu}}$ , under different polarization states: (b) upward polarization (*P*↑), (c) in-plane polarization (*P*→), and (d) downward polarization (*P*↓).

All of this demonstrates the enhanced effectiveness of the proposed descriptor in depicting the relationship between HER activity and the surface and electronic properties. It also manifests the importance of considering the influence of the amount of occupied *p* states for the latter. The strong correlations of the modified band edge also signal its applicability for different polarization states and its advantage over conventional band descriptors in illustrating the HER activity pattern for FE-based catalysts. Moreover, we know from previous surface adsorption analysis in Figure 3a–d that oxygen and TM dopants, in some cases, are not the directly relevant surface sites for the most stable hydrogen adsorption. The linear trend implies that there should be a neighboring electronic effect (possibly similar to the ligand effect) of TM doping that affects not only the O2*pz* band but also the TM3*dz* ^2^ band via coordination and charge transfer across the Fermi level upon doping, leading to varied surface reactivity of the oxygen or TM site.

In addition, it should also be noted that the geometrical effects, such as surface rumpling under each polarization state, are different, which also makes a difference to the surface electronic properties and activity trends. As previously observed in Figure S4, the effective polarization also shows periodic evolution with the atomic number in each TM group under varied polarization states. We explored the relationship between the effective polarization and the Δ*G*_H*_ for TM-doped BTO surfaces. As shown in Figure S11, for TM dopants, the correlation between *P*_eff_ and Δ*G*_H*_ exists yet varies with polarization states, and the *R* ^2^ values for the linear correlations follows the order: *P*_↓_ > *P*_→_ > *P*_↑_. Although the overall correlation for all TM dopants may be weak under certain polarization states, the correlation for each TM dopant group mostly shows a non-negligible correlation with coefficients above 0.50. This implies that the surface activity trends for TM-doped BTO are also affected by the effective polarization alone. The latter reflects the variation in surface and subsurface FE distortion along with the change in the local coordination environment in the [001] direction upon doping.

### ML Analysis

To gain a further understanding of the relationship between the surface catalytic activity, intrinsic dopant properties, and polarization states for TM-doped FE BTO, feature engineering techniques are used via ML to unfold the importance of the inherent properties that are independent of doped surface structures. In order to achieve this goal, we conduct analysis for 78 structures, including the undoped ones, with 26 structures for each polarization state. Reasonable feature selection is important for effective ML model training to grasp the hidden patterns underlying the data. To probe the relation between the HER activity of TM-doped FE BTO and surface-independent properties, we initially select a total of seven input parameters for the ML model, including one polarization parameter representing the initial polarization state of the BTO to be doped (P0, for simplicity, P0 = 1, 0, −1, where “1” represents upward polarization, “0” represents in-plane polarization, “–1” refers to downward polarization), as well as six commonly used atomic parameters, including the radii of TM atoms (TM_d), the electronegativity of TM atoms (TM_χ), the first and second ionization energies of TM atoms TM_I1, and TM_I2), the electron affinity of TM atoms (TM_A), and the number of outer electrons of TM atoms (TM_Ne). Among these 7 parameters, TM_d is an intrinsic physicochemical characteristic, while the rest (TM_χ, TM_I1, TM_I2, TM_A, and TM_Ne) are electronic properties. The Gradient Boosting Regression (GBR), Random Forest Regression (RFR), and Support Vector Machine (SVM) algorithms are adopted for ML and compared due to their advantages in dealing with limited data size.^39,83,84^ The input data for each parameter set are randomly divided into a training group and a testing group at a ratio of 7:3, followed by ML model training and testing.

Consequently, the ML method is found to fit well for the Δ*G*_H*_ (DFT) training and testing groups. As shown in Figure 6a–c, among them, the SVM ML model demonstrates a remarkably high correlation of linear fitting for Δ*G*_H*_ (ML) vs Δ*G*_H*_ (DFT) values for the training and testing groups, with *R* ^2^ values of 0.945 and 0.936, respectively. In other words, the ML-predicted Δ*G*_H*_ values are in decent agreement with the DFT results. The SVM ML model outperforms the GBR and RFR models for the test groups. This indicates that the as-obtained SVM ML model is more robust in predicting HER activity and should be suitable for further feature analysis. We also conducted 10-fold cross-validation^40^ for these ML models and found that the SVM shows smaller average mean absolute error (MAE) values, as shown in Table S3, demonstrating better generality and stability. Then, we carried out a permutation feature importance analysis for the ML model, which measures the reliance of a fitted ML model on each feature for a given data set with a single feature’s values randomly shuffled by observing the decreased accuracy of the ML model.^85^ As presented in Figure 6d, the number of outer electrons of the TM dopant (TM_Ne) accounts for the largest feature importance, as high as 1.0, suggesting that it is a dominant feature in ML prediction. The second ionization energy of the TM dopant (TM_I2) shows a lower importance of 0.36, while the importance of P0 (initial polarization state) is 0.20, which is relatively low yet non-negligible.

**Figure 6:**
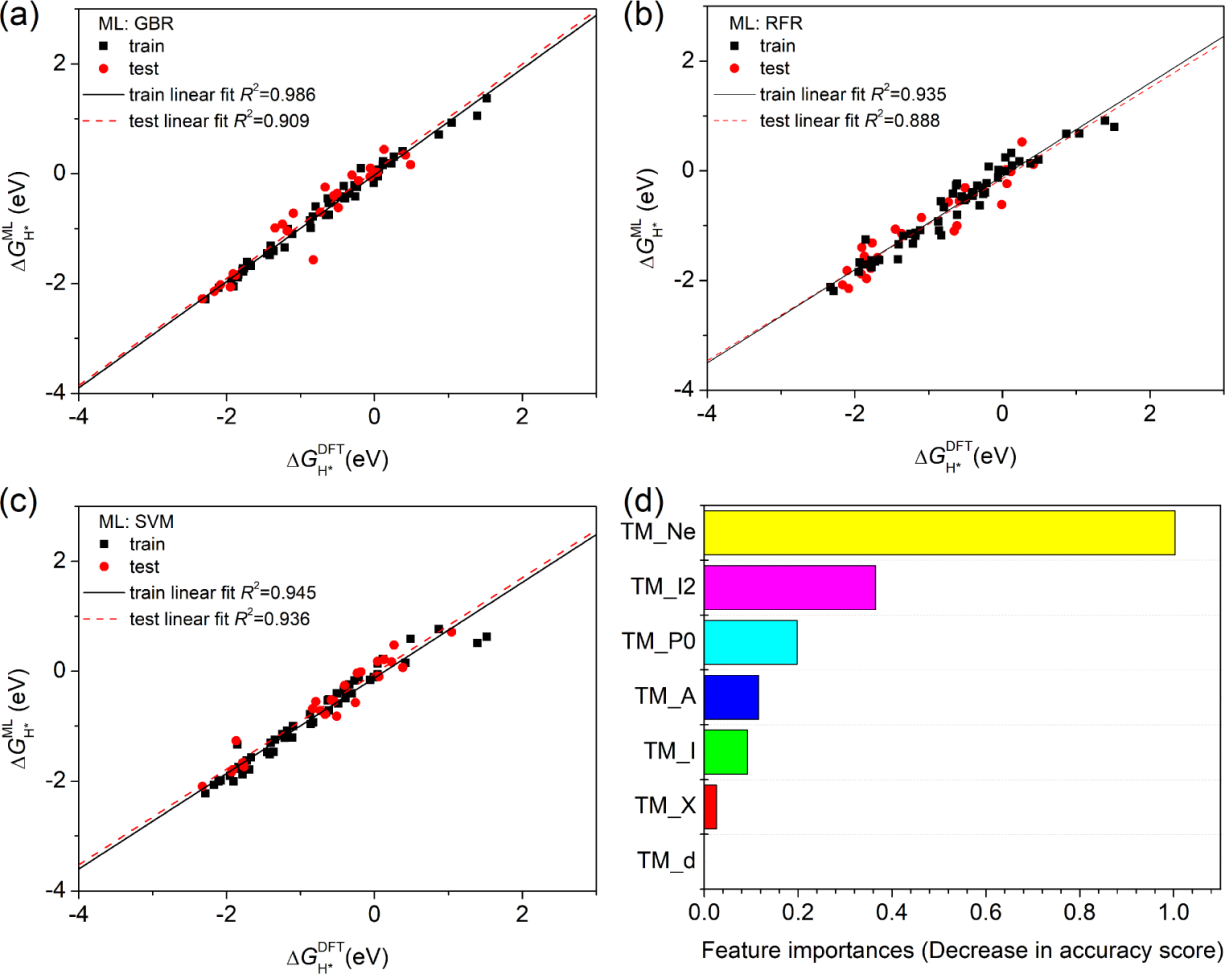
DFT-calculated and ML-predicted Δ*G*_H*_ values for train and test data and corresponding linear regressions for different ML models of (a) GBR, (b) RFR, and (c) SVM. (d) Feature importance for various input parameters of the as-obtained SVM ML model.

The other four features exhibit quite low feature importance and hence are much less predictive in ML. The reason why the number of outer electrons is notably predictive in ML may be linked to the TM dopant-dependent charge transfer behavior upon hydrogen adsorption, as indirectly reflected by the shift of the antibonding energy center and the amount of occupied oxygen *p*_*z*_ states mentioned above. This atomic property was also reported to show a high correlation with hydroxyl adsorption energies in oxygen evolution/reduction electrocatalysis.^86^ The present ML analysis could be useful for accelerating the screening of doped FE electrocatalysts in the future.

## Conclusion

We computationally investigate the TM doping impact on the HER activity of FE BTO and identify optimal dopants for HER based on DFT calculations and ML. We find that FE BTO doped by the first several TM elements in each *d* group possesses higher synthesizability, while most TM-doped surfaces are electrochemically stable. We find that several early-to-middle dopants in each group show favorable hydrogen adsorption energetics toward HER, while the Mo-doped BTO surface exhibits optimal HER activity under all polarization states. We reveal that the hydrogen adsorption strength on doped surfaces is directly correlated with the shift of the antibonding energy center of the hydrogen bond of varied geometry on different sites. Interestingly, we propose a novel electronic fingerprint based on the upper band edge of the surface oxygen 2*p* band before hydrogen adsorption. This indirect fingerprint outperforms the conventional *p*-band center and band edge in describing the HER activity trend of TM-doped surfaces for different polarization states. Through ML analysis, we establish an implicit link between HER activity and surface-independent properties, including the intrinsic TM dopant atomic properties and the polarization state. The established SVM ML model is tested with remarkable predictive accuracy for HER activity. The number of outer electrons of the TM dopant is found to be the most dominant feature in the as-obtained ML model for HER activity prediction. Our findings can be enlightening to understand TM-doped FE catalysts and accelerate the design and discovery toward the HER and beyond.

## Supplementary Material



## Notes

Δ*G*_H*_ on BTO surfaces doped by optimal TM dopants with and without implicit solvent; Δ*G*_H*_ on Mo-doped BTO *p*(2×2); 10-fold cross validation for various ML models; stable chemical potential region at varied Δμ_O_; formation energy trends of TM-doped BTO at varied *Δ*μ_O_; grand potential phase diagrams; the effective polarization and PDOS; surface sites on typical TM-doped surfaces; Pourbaix diagrams for optimal dopants; structures of Mo-doped BTO *p*(2×2) surface with hydrogen; the dependence of Δ*G*_H*_ on ICOHP; correlation between Δ*G*_H*_ and ε̅_abd_; correlation between Δ*G*_H*_ and ε*®_p_* and $\varepsilon_{u}$ ; correlation between Δ*G*_H*_ and effective polarization; correlation of Δ*G*_H*_ with surface TM *dz* ^2^ band center, ${\mathrm{\varepsilon ̅}}_{dz^{2}}$ ; and correlation of Δ*G*_H*_ with surface work function (PDF)
