# Supplementary material for: DFT-Machine Learning Joint Exploration of Transition Metal-Doped Ferroelectric BaTiO3 for Electrocatalytic Hydrogen Evolution
Source: ACS Appl Mater Interfaces. 2025 Jun 4;17(24):35396–408. doi: 10.1021/acsami.5c02406 (PMC12186225; doi:10.1021/acsami.5c02406)
Supplement: Supplementary file 1 [file am5c02406_si_001.pdf]

Supporting Information

DFT-Machine Learning Joint Exploration of Transition-Metal-Doped Ferroelectric $BaTiO_3$ for Electrocatalytic Hydrogen Evolution

Haifa Qiu [a,b], Ming Yang [a,c*], Haitao Huang [a,b*]

[a] *Department of Applied Physics, The Hong Kong Polytechnic University, Hung Hom, Kowloon, Hong Kong, China*

[b] *Research Institute for Smart Energy, The Hong Kong Polytechnic University, Hung Hom, Kowloon, Hong Kong, China*

[c] *Research Centre for Nanoscience and Nanotechnology, The Hong Kong Polytechnic University, Hung Hom, Kowloon, Hong Kong, China*

* Email: aphhuang@polyu.edu.hk; kevin.m.yang@polyu.edu.hk

Table S1 Gibbs free energy change of hydrogen adsorption, ΔG_{H*}, on BTO surfaces doped by optimal TM dopants with and without implicit solvent.

Polarization state	Optimal TM dopant	ΔG_{H*} (without implicit solvent)/eV	ΔG_{H*} (**with implicit solvent**)/eV
$P\uparrow$	Cr	-0.059	**-0.053**
	Nb	0.119	**0.200**
	**Mo**	**-0.054**	**0.063**
	Ru	-0.010	**-0.076**
	Ta	-0.185	**-0.077**
$P\rightarrow$	V	0.039	**0.238**
	**Mo**	**0.045**	**0.089**
	Ta	0.041	**0.095**
$P\downarrow$	V	0.115	**0.244**
	**Mo**	**0.129**	**0.200**

Table S2 Gibbs free energy change of hydrogen adsorption, ΔG_{H*}, of Mo-doped BTO $p(2 \times 2)$ surface under varied polarization states.

	$P_{\uparrow}$	$P_{\rightarrow}$	$P_{\downarrow}$
$c(2 \times 2)$	-0.054	-0.044	0.129
$p(2 \times 2)$	-0.244	-0.012	0.231

Table S3 10-fold cross validation for different ML models of GBR, RFR, and SVM. (MAE: mean absolute error, MDAE: median absolute error)

Fold number	MAE/MDAE (eV)		
	**GBR**	**RFR**	**SVM**
1st	0.28972212 / 0.27327844	0.25159152 / 0.25161205	0.18225613 / 0.17162774
2nd	0.42480575 / 0.42589405	0.39002741 / 0.46775195	0.32478584 / 0.31318393
3rd	0.43150262 / 0.5138875	0.42002856 / 0.45025385	0.21242506 / 0.1677967
4th	0.20044549 / 0.10035539	0.26971275 / 0.1656218	0.25772859 / 0.15854786
5th	0.16936078 / 0.08831015	0.22993969 / 0.2294909	0.17785772 / 0.15155868
6th	0.21926628 / 0.191536	0.22814442 / 0.2663716	0.17470227 / 0.19040506
7th	0.27490872 / 0.28843685	0.30760696 / 0.30754775	0.18225613 / 0.27290093
8th	0.33779358 / 0.09739219	0.35030075 / 0.3110294	0.27133336 / 0.2841641
9th	0.49517905 / 0.15328913	0.45404627 / 0.1911419 /	0.42309415 / 0.23528128
10th	0.50140337 / 0.38455707	0.55920394 / 0.4753285	0.38775522 / 0.16437399
10-fold Average	0.334 / 0.252	0.346 / 0.311	**0.266 / 0.211**

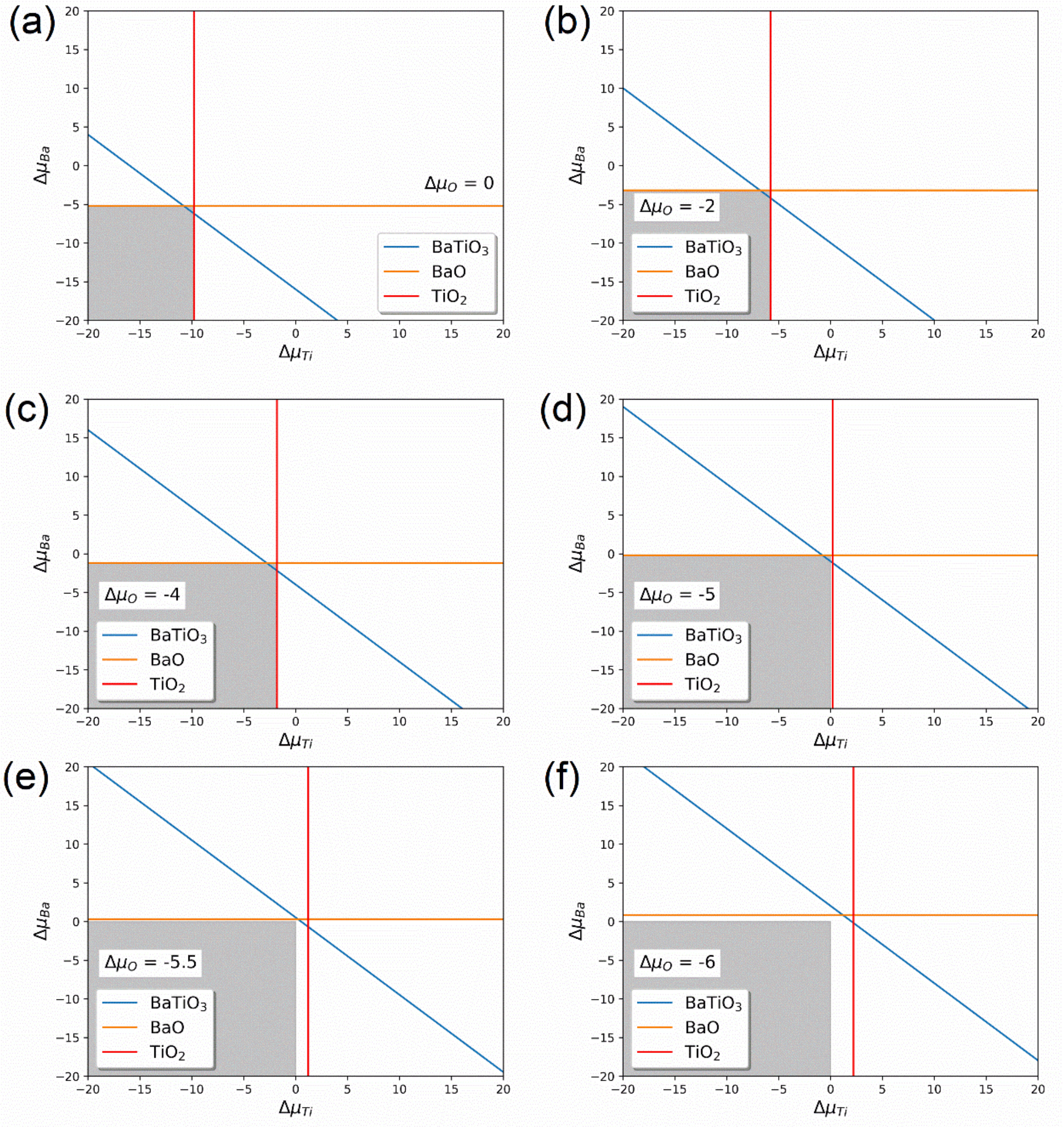

Figure S1 The evolution of stable chemical potential region with varied oxygen chemical potentials of (a) 0 eV, (b) -2 eV, (c) -4 eV, (d) -5 eV, (e) -5.5 eV, and (f) -6 eV. The shaded area refers to the stable chemical potential region for metallic Ba and Ti, beyond which Ba or Ti will precipitate.

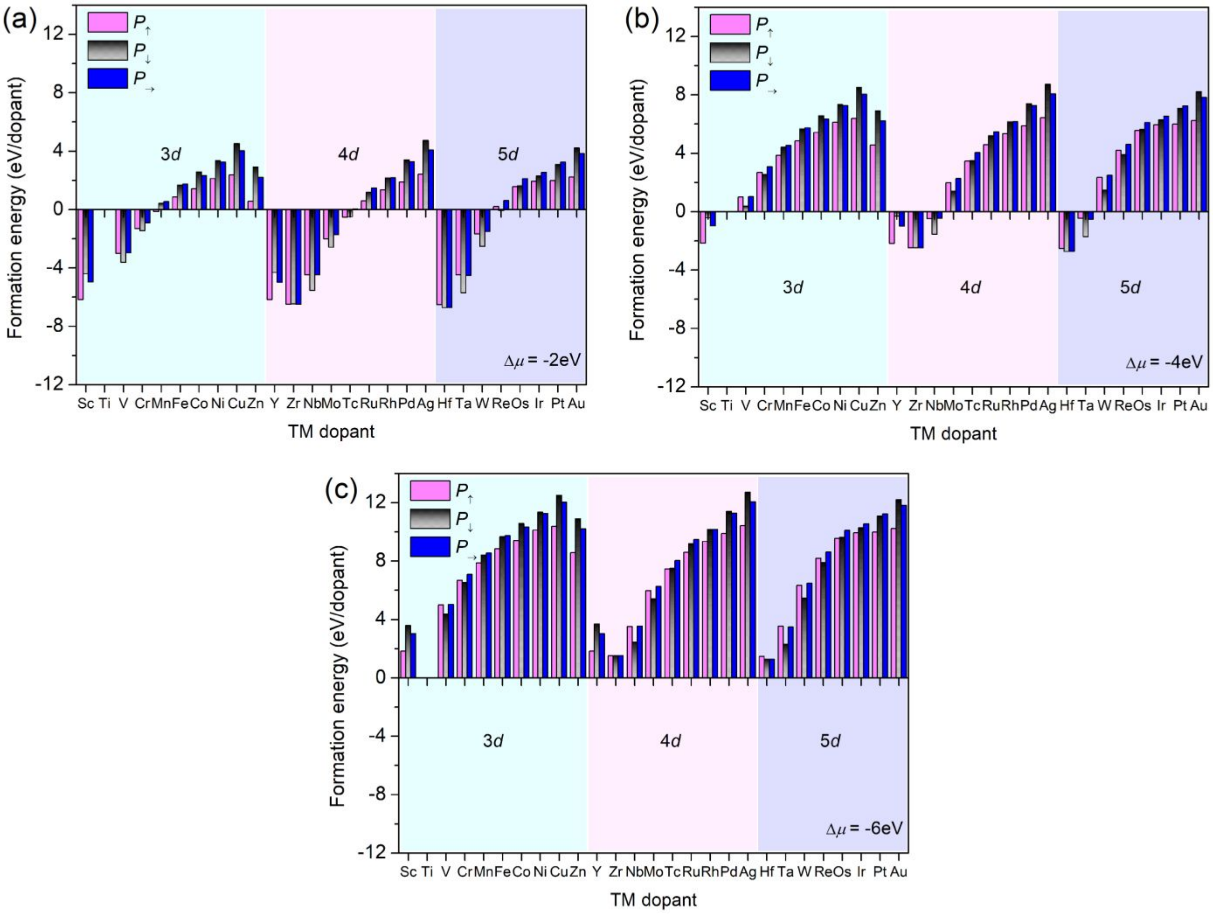

Figure S2 The formation energy of TM-doped FE BTO surfaces at various oxygen chemical potentials of (a) -2 eV, (b) -4 eV, and (c) -6 eV under different polarization states.

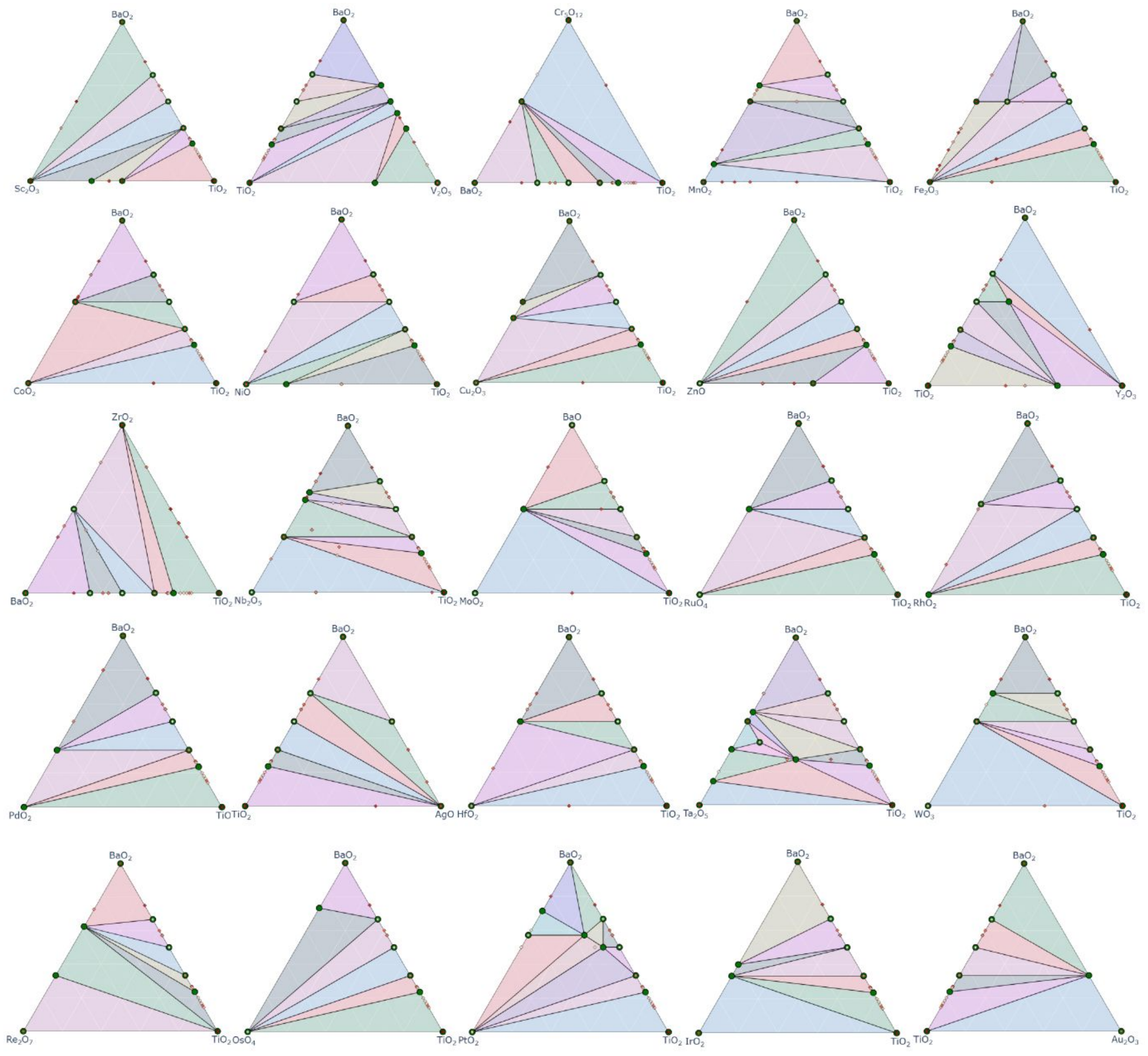

Figure S3 Grand potential phase diagram of ternary transition metal oxide systems for TM-doped BTO derived from Pymatgen program and Materials Project[1-4]. The temperature and oxygen chemical potential for each phase diagram of TM dopant is 300K and 0 eV, respectively, unless otherwise specified. For Mo, the temperature and oxygen chemical potential is 1000K and -2 eV, respectively. For Ag, the temperature is 500K.

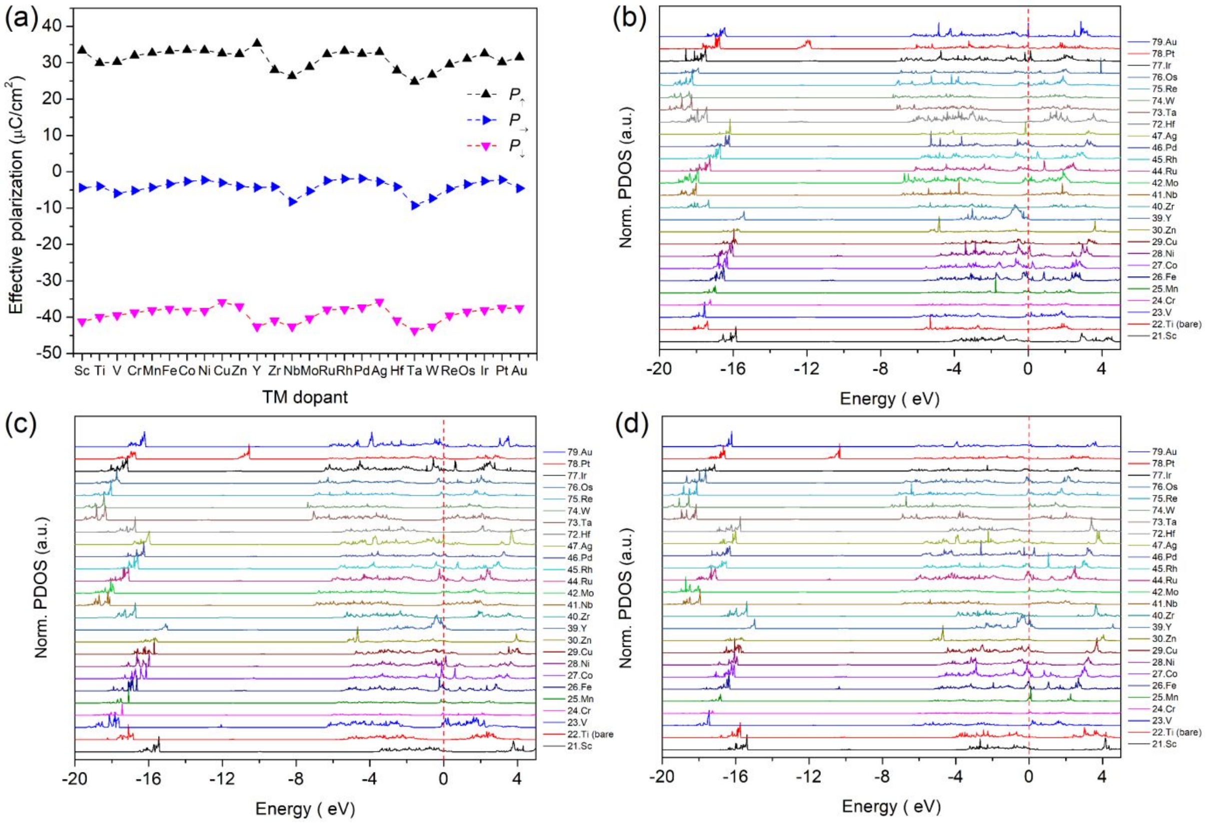

Figure S4 Structural and electronic reconstruction upon TM doping. (a) The evolution of effective polarization for various TM-doped BTO under different polarization states. The PDOS of TM-doped surface for various TM dopants under (b) upward, (c) in-plane, and (d) downward polarization states. Red dashed line indicates the Fermi level.

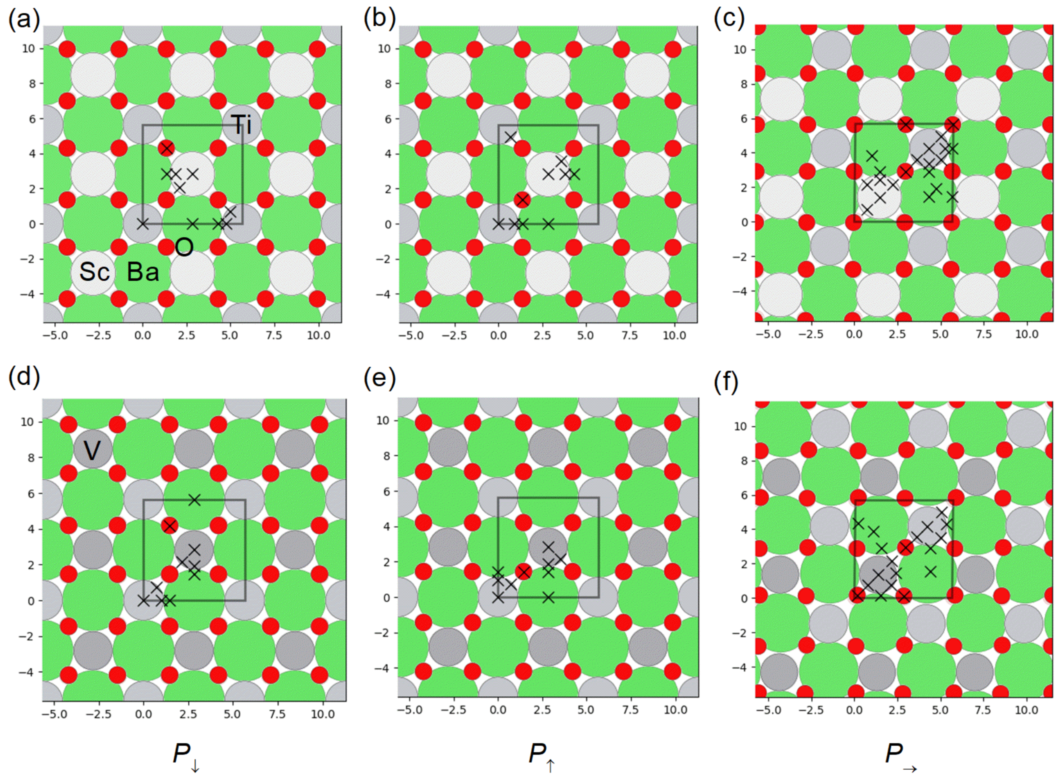

Figure S5 The irreducible surface sites on typical (a-c) Sc-doped and (d-f) V-doped FE BTO surfaces before hydrogen adsorption under (a,d) downward, (b,e) upward, and (c,f) in-plane polarization states. The black cross in the square indicates the surface adsorption site. Atom names are labelled in Figure S5a,d

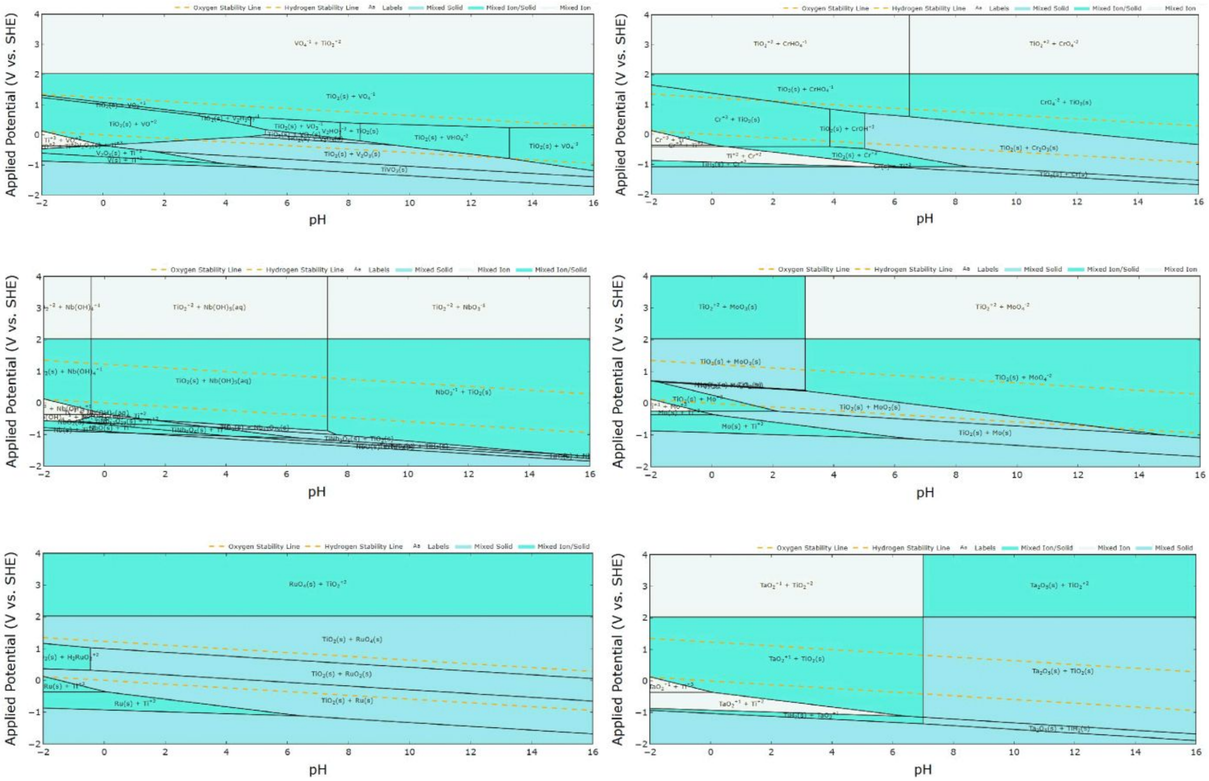

Figure S6 Pourbaix diagram for TM-doped TiO_2 systems at applied potential and pH for optimal dopants (V, Cr, Nb, Mo, Ru, Ta) for reference.

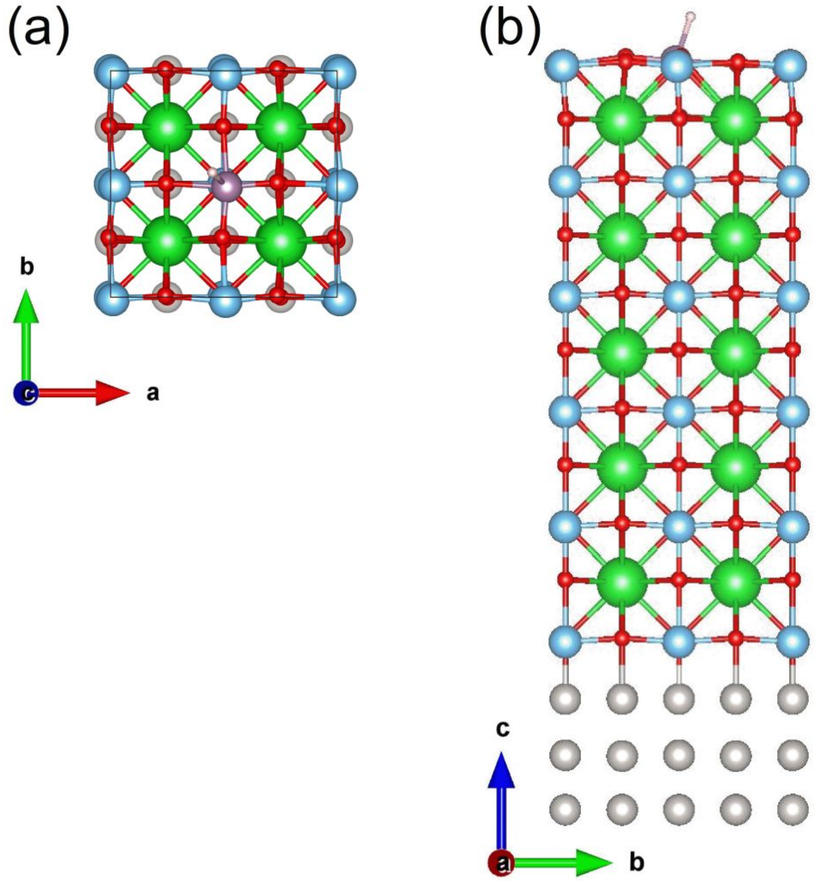

Figure S7 (a) The top view from [001] direction and (b) side view from [100] direction of the Mo-doped BTO $p(2 \times 2)$ surface upon hydrogen adsorption under the downward polarization state. The Pt, Ba, Ti, O, Mo atoms are in grey, green, blue, red, and purple color, respectively.

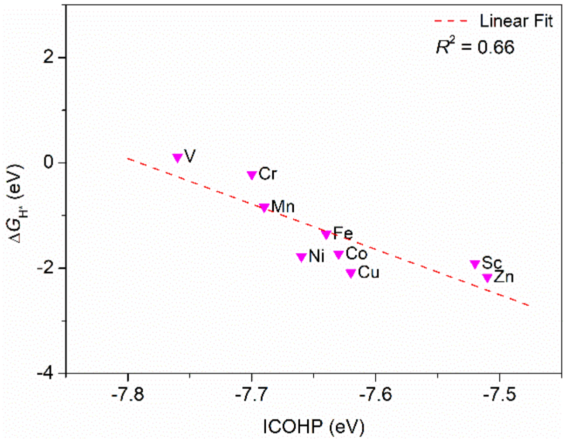

Figure S8 The dependence of Gibbs free energy change of hydrogen adsorption, ΔG_{H^*}, with ICOHP values for 3*d* TM-doped BTO surfaces under downward polarization state.

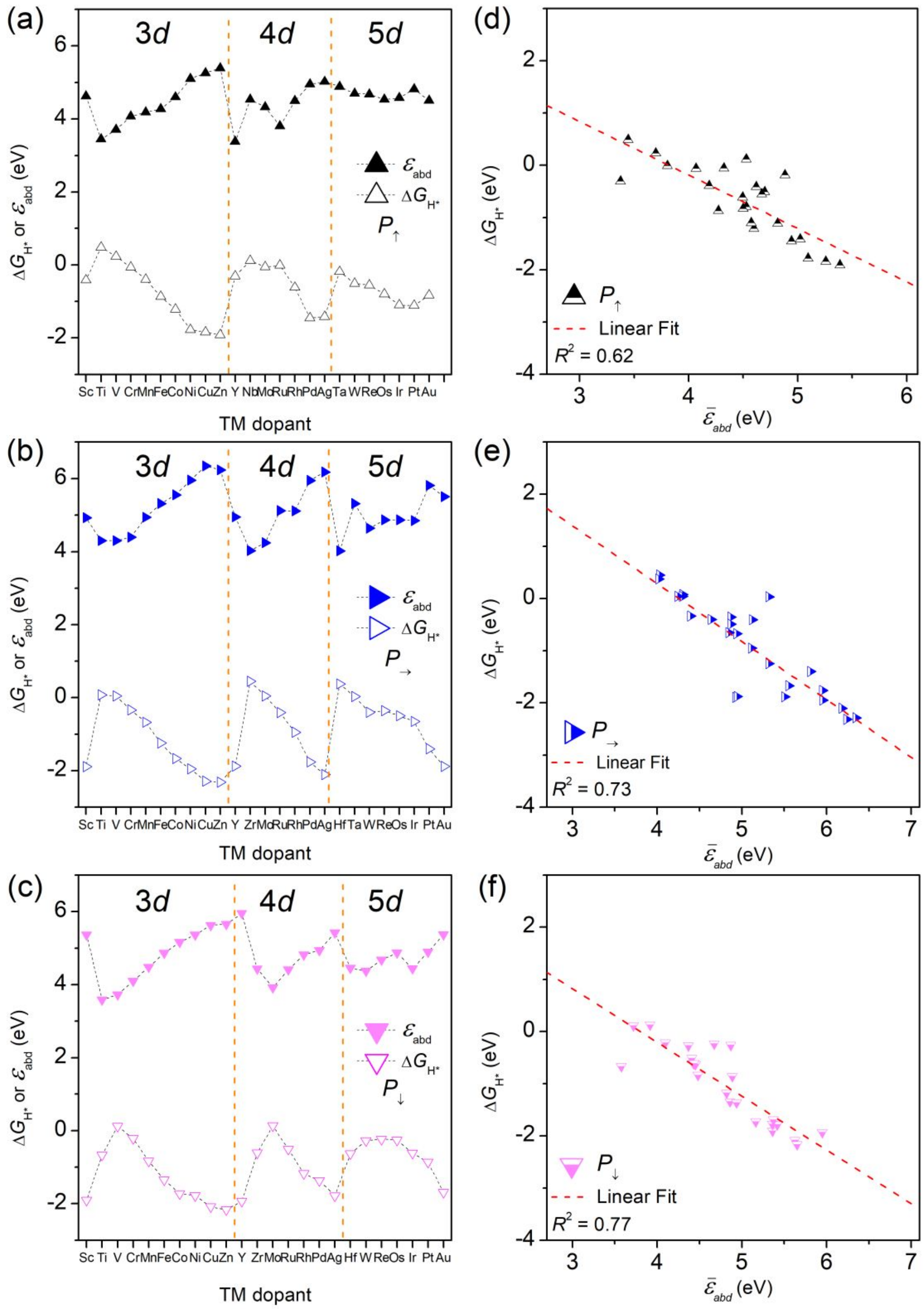

Figure S9 (a-c) The evolution of Gibbs free energy change of hydrogen adsorption, ΔG_{H*}, and averaged antibonding band center, $\bar{\varepsilon}_{abd}$, and (d-f) corresponding linear fitting for TM-doped BTO surface under varied polarization states. One or two TM dopants are not included in the linear regression under upward (Zr, Hf), in-plane (Nb), and downward (Nb, Ta) polarization states.

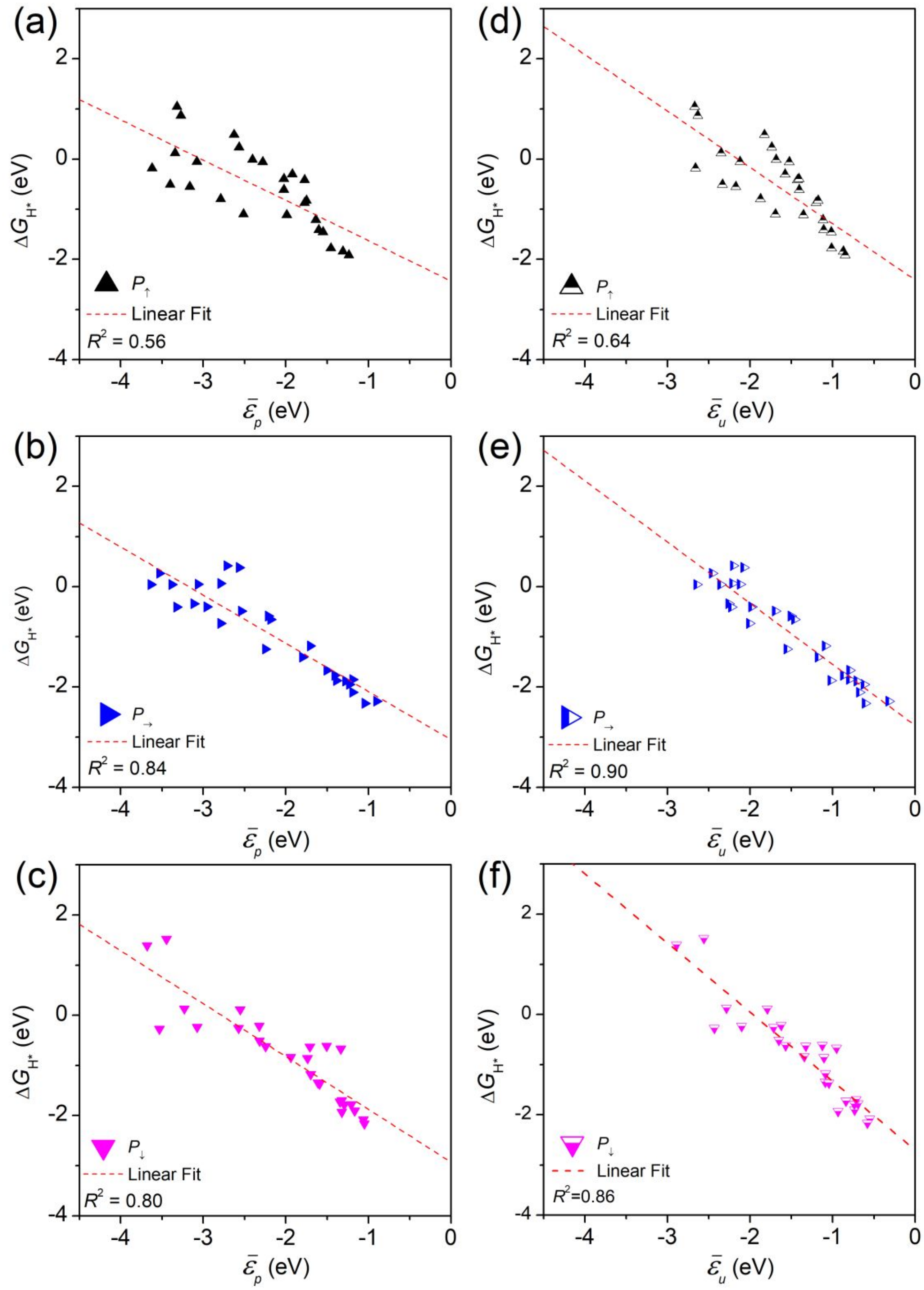

Figure S10 The correlation of Gibbs free energy change of hydrogen adsorption, ΔG_{H^*}, with (a-c) surface oxygen $2pz$ band center, $\bar{\varepsilon}_p$, and (d-f) surface oxygen $2pz$ band edge, ε_u, for TM-doped BTO surface under varied polarization states.

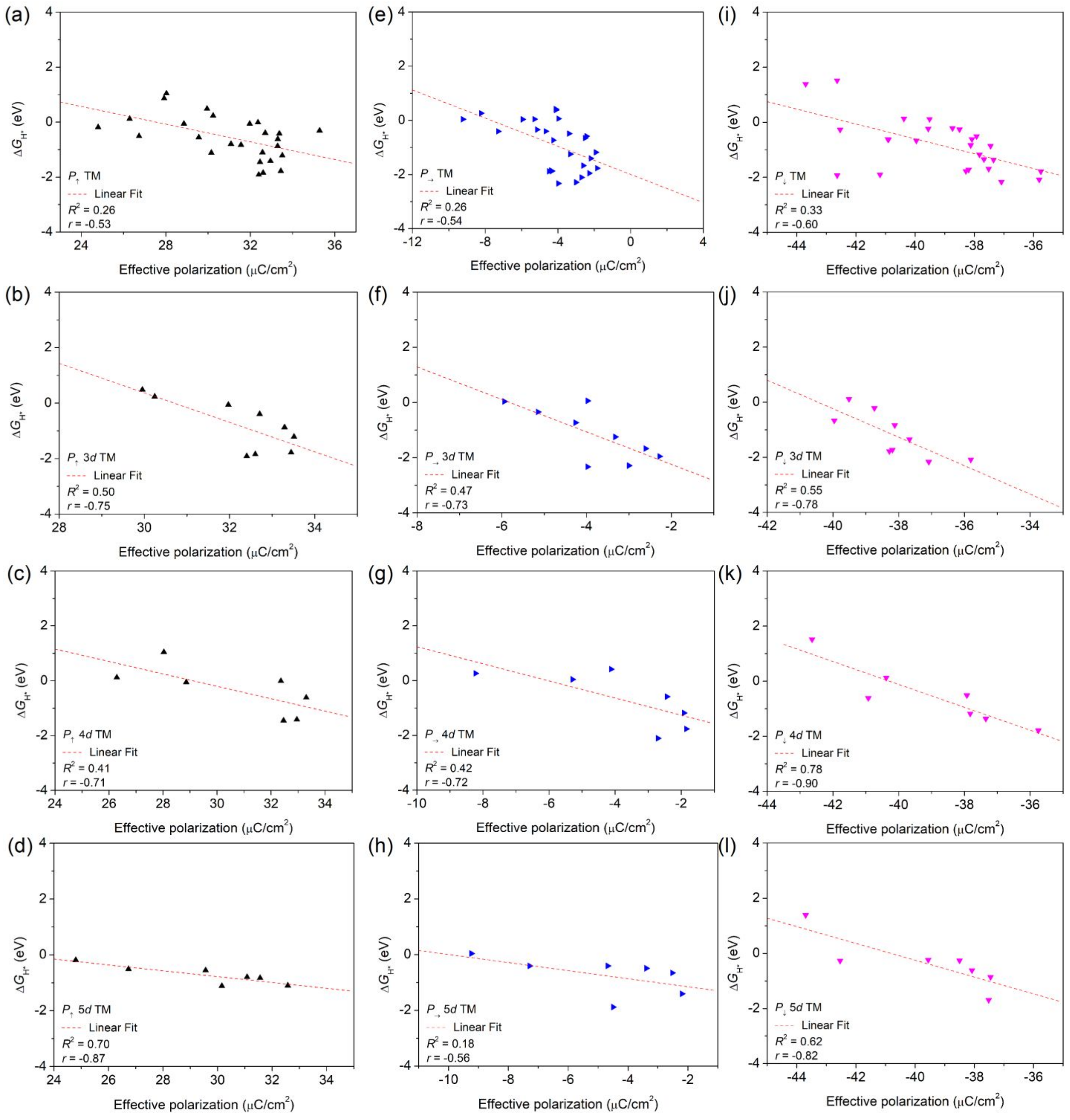

Figure S11 The correlation between the Gibbs free energy change of hydrogen adsorption, ΔG_{H^*}, and the effective polarization of TM-doped BTO surface under (a-d) upward polarization state, (b-h) in-plane polarization state, and (c-l) downward polarization state.

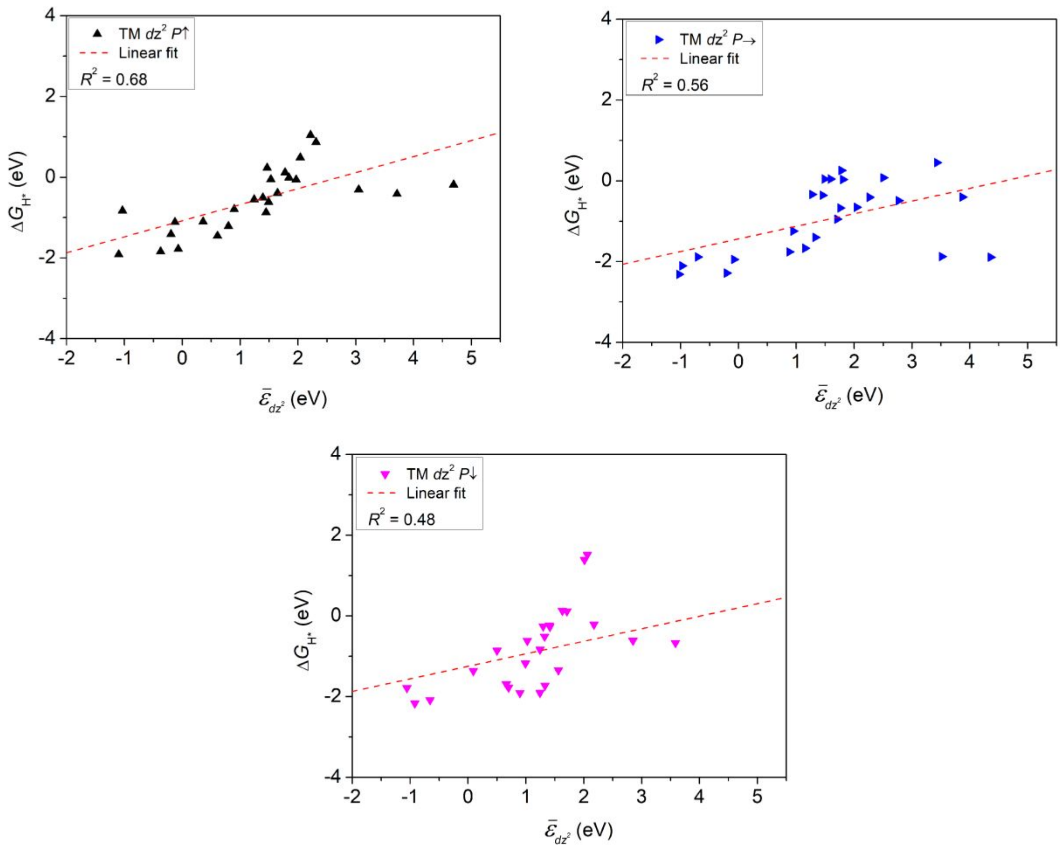

Figure S12 The correlation of Gibbs free energy change of hydrogen adsorption, ΔG_{H^*}, with surface TM dz^2 band center, $\bar{\varepsilon}_{dz^2}$, for TM-doped BTO surface under varied polarization states.

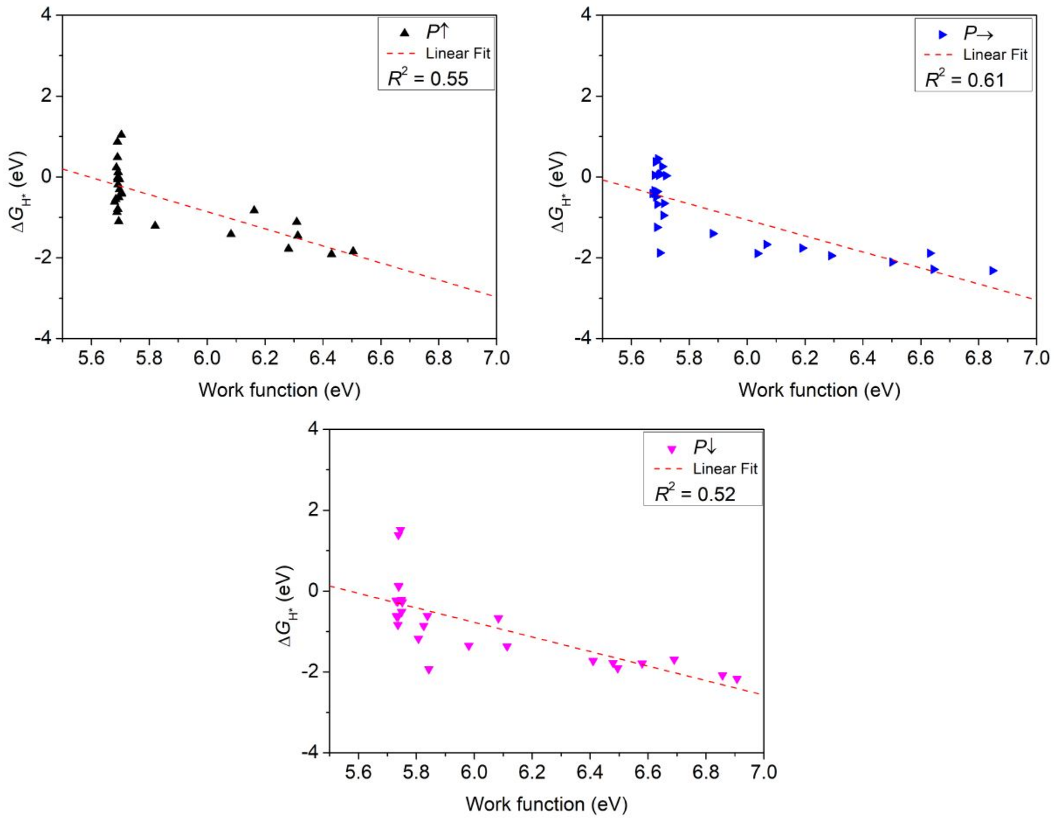

Figure S13 The correlation of Gibbs free energy change of hydrogen adsorption, ΔG_{H^*}, with surface work function for TM-doped BTO surface under varied polarization states.

REFERENCES

(1) Jain, A.; Ong, S. P.; Hautier, G.; Chen, W.; Richards, W. D.; Dacek, S.; Cholia, S.; Gunter, D.; Skinner, D.; Ceder, G.; Persson, K. A. Commentary: The Materials Project: A Materials Genome Approach to Accelerating Materials Innovation. *APL Mater.* **2013**, *1*, 011002.

(2) Patel, A. M.; Nørskov, J. K.; Persson, K. A.; Montoya, J. H. Efficient Pourbaix Diagrams of Many-Element Compounds. *Phys. Chem. Chem. Phys.* **2019**, *21*, 25323-25327.

(3) Singh, A. K.; Zhou, L.; Shinde, A.; Suram, S. K.; Montoya, J. H.; Winston, D.; Gregoire, J. M.; Persson, K. A. Electrochemical Stability of Metastable Materials. *Chem. Mater.* **2017**, *29*, 10159-10167.

(4) Persson, K. A.; Waldwick, B.; Lazic, P.; Ceder, G. Prediction of Solid-Aqueous Equilibria: Scheme to Combine First-Principles Calculations of Solids with Experimental Aqueous States. *Phys. Rev. B* **2012**, *85*, 235438.
